# Comparison between watchful waiting strategy and early initiation of renal replacement therapy in the critically ill acute kidney injury population: an updated systematic review and meta-analysis

**DOI:** 10.1186/s13613-020-0641-5

**Published:** 2020-03-03

**Authors:** Jia-Jin Chen, Cheng-Chia Lee, George Kuo, Pei-Chun Fan, Chan-Yu Lin, Su-Wei Chang, Ya-Chung Tian, Yung-Chang Chen, Chih-Hsiang Chang

**Affiliations:** 1 Department of Nephrology, Linkou Chang Gung Memorial Hospital, Taoyuan, Taiwan; 2 Department of Nephrology, Kidney Research Center, Linkou Chang Gung Memorial Hospital, No 5 Fu-shin Street, Taoyuan, 333 Taiwan; 3 Clinical Informatics and Medical Statistics Research Center, College of Medicine, Chang Gung University, Taoyuan, Taiwan; 4 Division of Critical Care Nephrology, Department of Nephrology, Chang Gung Memorial Hospital, Keelung, Taiwan

**Keywords:** Renal replacement therapy, Timing, Acute kidney injury, Watchful waiting strategy

## Abstract

**Background:**

The optimal timing of renal replacement therapy (RRT) initiation is debatable. Many articles in this field enrolled trials not based on acute kidney injury. The safety of the watchful waiting strategy has not been fully discussed, and late RRT initiation criteria vary across studies. The effect of early RRT initiation in the AKI population with high plasma neutrophil gelatinase-associated lipocalin (NGAL) has not been examined yet.

**Methods:**

In accordance with PRISMA guidelines, the PubMed, Embase, and Cochrane databases were systemically searched for randomized controlled trials (RCTs). Trials not conducted in the AKI population were excluded. Data of study characteristics, primary outcome (all-cause mortality), and related secondary outcomes [mechanical ventilation (MV) days, length of hospital stay, RRT days, and length of ICU stay] were extracted. The outcomes were compared between early and late RRT groups by estimating the pooled odds ratio (OR) for binary outcomes and the weighted mean difference for continuous outcomes. Prospective trials were also examined and analyzed using the same method.

**Results:**

Nine RCTs with 1938 patients were included. Early RRT did not provide a survival benefit (pooled OR, 0.88; 95% confidence interval [CI] 0.62–1.27). However, the early RRT group had significantly fewer MV days (pooled mean difference, − 3.98 days; 95% CI − 7.81 to − 0.15 days). Subgroup analysis showed that RCTs enrolling the surgical population (*P* = .001) and the AKI population with high plasma NGAL (*P* = .031) had favorable outcomes regarding RRT days in the early initiation group. Moreover, 6 of 9 RCTs were selected for examining the safety of the watchful waiting strategy, and no significant differences were found in primary and secondary outcomes between the early and late RRT groups.

**Conclusions:**

Overall, early RRT initiation did not provide a survival benefit, but a possible benefit of fewer MV days was detected. Early RRT might also provide the benefit of shorter MV or RRT support in the surgical population and in AKI patients with high plasma NGAL. Depending on the conventional indication for RRT initiation, the watchful waiting strategy is safe on the basis of all primary and secondary outcomes.

## Background

Acute kidney injury (AKI) is a frequent problem in critically ill patients, and it is associated with high morbidity and mortality. Even though improved renal replacement therapy (RRT) techniques and enhanced knowledge of critical care have increased the survival of critically ill patients with AKI in recent decades [1], recent research still shows a mortality rate of 24% in the AKI population [2], with 19% of the critically ill AKI population requiring RRT [3]. More than half of critically ill patients have AKI, and mortality is worse with progressive AKI [4]. Over the last 2 decades, the optimal timing of RRT initiation and the role of early renal support have been widely discussed. Earlier retrospective and prospective studies revealed the survival benefits of early RRT initiation; however, since the AKIKI and ELAIN trials published in 2016, these survival benefits have become debatable. In recent years, several meta-analyses have been published, most of which investigated prospective or retrospective studies owing to the lack of large-scale randomized controlled trials (RCTs), and in these meta-analyses, a definitive conclusion regarding the optimal time of RRT initiation could not be drawn [5–11]. Conventional indications (refractory fluid overload, acidemia, hyperkalemia, and uremic complication) for RRT initiation are indisputable over several years. Moreover, bedside intensivists or consulting nephrologists often use conventional indications as well as the patient’s clinical condition to make decisions regarding RRT initiation. Several meta-analyses included studies that did not use conventional indications as late criteria (most prospective trials) [12] or studies that were not based on the AKI population [13–15]. To the best of our knowledge, therefore, little is known regarding the safety of the watchful waiting strategy and conventional indications for initiating RRT in AKI patients without the emergent need for RRT initially. The safety concern involves the risk of exposing critically ill patients to AKI-related complications (severe fluid overload, uremia, hyperkalemia, and metabolic acidosis). Furthermore, recent advancements in novel biomarkers (neutrophil gelatinase-associated lipocalin [NGAL], cystatin, calprotectin, and [insulin-like growth factor-binding protein 7] × [tissue inhibitor of metalloproteinase 2]) may provide insights into AKI patient triage, accelerating AKI or acute tubular necrosis (ATN) diagnosis and improving AKI prognosis and RRT prediction [16–22]. Moreover, biomarker-guided AKI intervention may lead to improved outcomes [23]. A higher NGAL level is now considered a marker of poor prognosis. According to Bagshaw et al., some severely critically ill patients are unlikely to benefit from early RRT, and several potential drawbacks should be considered, such as higher costs or unnecessary complications from dialysis [74]. Several studies in this field used plasma NGAL as their enrollment criterion. Nevertheless, no meta-analysis has discussed whether the early initiation of RRT has positive or negative effects in the high-risk AKI population with high plasma NGAL.

We attempted to supplement previous findings by including three recent RCTs (EARLY-RRT, 2018 [59]; FST trial, 2018 [58]; and IDEAL-ICU, 2018 [57]) in this meta-analysis to examine the effect of early RRT initiation in the AKI population with high plasma NGAL, and we assessed the safety of the watchful waiting strategy in this population.

## Methods

### Literature search

Two investigators (JJ-C and CH-C) systematically and independently conducted a review of published data in accordance with Preferred Reporting Items for Systematic Reviews and Meta-Analyses (PRISMA) guidelines to investigate the effect of the timing of RRT initiation on outcomes in patients with AKI. A search of the PubMed, Embase, and Cochrane electronic databases was performed to identify all relevant studies published from January 2000 to March 2019. The search was restricted to RCTs or prospective cohort studies conducted in adult patients and published in the English language. The following keywords were used: acute kidney injury, acute renal failure, acute kidney failure, renal replacement therapy, timing, hemodialysis, dialysis, hemofiltration, continuous renal replacement therapy, time to treatment, time, early, late, and accelerate. Studies published before 2000 were not included in this study because advancements in AKI knowledge and RRT technology would have resulted in improvement in AKI prognosis. The detailed search strategy is provided in Additional file 1: Table S1.

### Study selection

Study inclusion eligibility was evaluated by browsing the titles/abstracts and subsequently the full texts of published articles. Any differences in opinion regarding study eligibility for inclusion were resolved through discussion. Full texts of potentially relevant articles were retrieved online. Studies were included if they met the following criteria:

1. Adult human study.
2. AKI (either guideline-based AKI criteria, such as risk, injury, failure, loss of kidney function, and end-stage kidney disease [RIFLE] [24], Acute Kidney Injury Network [25], Kidney Disease: Improving Global Outcomes [KDIGO] [26], or predefined AKI criteria of individual studies).
3. Comparison of the effect of early versus late RRT on the relevant primary outcome (mortality) and secondary outcomes (intensive care unit [ICU] length of stay [LOS], hospital LOS, mechanical ventilation [MV] time, RRT duration, and RRT dependence).
4. Clear definitions of early and late RRT initiation.

Studies with insufficient information (no actual event numbers) or those not based on the AKI population were excluded. Review articles and meta-analyses were not included for analysis, but their citations and references were searched to explore additional relevant studies.

### Data extraction

Data of study characteristics that were extracted included first author, year of publication, study location, study design, AKI definition, inclusion and exclusion criteria, patient number (total population and early and late RRT initiation groups separately), patient population (medical, surgical, or mixed), RRT modality, whether patients with the emergent need of RRT were excluded before randomization or grouping, early and late RRT initiation criteria, and whether NGAL was used as an enrollment criterion (either for ATN diagnosis or for AKI patient risk triage). All these data were extracted and summarized (Additional file 1: Tables S2, S3). The primary outcome was mortality (including in-hospital mortality and 30-, 60-, or 90-day mortality). Secondary outcomes were ICU or hospital LOS; RRT dependence, RRT duration, and RRT-free days during the follow-up period; MV time or MV-free days during the follow-up period; RRT dependence; and renal recovery (Additional file 1: Tables S2, S3). The following parameters at the time of RRT initiation or randomization were extracted: serum creatinine, urea, disease severity score (Acute Physiology and Chronic Health Evaluation II [APACHE II] score and Sequential Organ Failure Assessment [SOFA] score), age, vasopressor use, septic shock/sepsis, and MV use. Differences in baseline patient condition between early and late RRT initiation groups are summarized in Additional file 1: Tables S3, S4.

### Assessment of evidence quality and risk of bias

The risk of bias in the included RCTs was assessed independently by two reviewers (JJ-C and CH-C) using Cochrane Collaboration’s tool for assessing the risk of bias [27]. The risk-of-bias tool covers six domains of bias: selection bias, performance bias, detection bias, attrition bias, reporting bias, and other bias. Each study was reviewed and rated as having a high, low, or unclear risk of bias as per the Cochrane Handbook for Systematic Reviews of Interventions version 5.1 (online) [67]. The quality of evidence for primary and secondary outcomes of this meta-analysis was assessed using the Grading of Recommendations Assessment, Development and Evaluation (GRADE) Working Group methodology. We summarized the results in tables constructed using the GRADEpro online tool [69].

### Definition of early versus late RRT initiation

The definitions for early and late RRT initiation were diverse among studies. A detailed explanation is provided in Additional file 2: Document S1.

### Outcome measures

#### Primary outcome

The primary outcome of interest was all-cause mortality. In-hospital mortality and 30-, 60-, 90-, and 180-day mortality were recorded. The total patient numbers and the number of deaths in the early and late RRT initiation groups were recorded. The overall mortality was analyzed if 60- to 90-day mortality was available. Otherwise, we extracted data in the following sequence: in-hospital mortality, 30-day mortality, and 180-day mortality.

#### Secondary outcomes

The secondary outcomes were ICU LOS, hospital LOS, RRT duration, or RRT-free days during the follow-up period; MV time or MV-free days during the follow-up period; RRT dependence; and renal recovery. For continuous outcomes, the mean with standard deviation or the median with interquartile range was extracted. Under the assumption of normal distribution, we transformed the interquartile range into standard deviation according to the Cochrane Handbook for Systematic Reviews of Interventions [70]. Some studies reported MV-free days or RRT-free days at a fixed time interval (e.g., 60 days); subsequently, MV-free days or RRT-free days were subtracted from the fixed time interval to calculate the duration. The definition of renal recovery varied substantially across studies, and one study did not report the definition of renal recovery [28]. The 60-day RRT dependence was analyzed first; if it was not available, we used reported information in the following sequence: 28-day and 90-day RRT dependence.

### Statistical analysis

In this meta-analysis, the pooled odds ratio (OR) for binary outcomes and the weighted mean difference for continuous outcomes were calculated. The data from individual studies were pooled using the random effect model. We conducted subgroup analysis to examine the possible modification effects of the patient population (medical, surgical, or mixed), study design (whether patients with the emergent need for RRT were excluded before enrollment or randomization in the trial), and the AKI population with or without high plasma NGAL. We performed subgroup analysis using the mixed effect model to study four outcomes: death, ICU LOS, MV days, and RRT days. Heterogeneity between studies was assessed using the *I*^2^ statistic. In addition, to evaluate some of the results of meta-analysis that may be greatly affected by the result of one outlier study, we performed a cumulative meta-analysis.

To assess the safety of the watchful waiting strategy and conventional indications for RRT in the critically ill AKI population, each included RCT was examined for predefined criteria:

1. Exclusion of patients with the emergent need for RRT before randomization.
2. Equal baseline condition and disease severity (assessed using APACHE II, SOFA, Simplified Acute Physiology Score II, age, vasopressor use, MV, sepsis/shock, and baseline renal function).
3. Conventional indications for RRT as late criteria.

Studies that fulfilled all the aforementioned criteria were evaluated through sensitivity analysis. The primary outcomes of the watchful waiting strategy and the significance of secondary outcomes were validated in the context of information size and effect size by using Trial Sequential Analysis (TSA) with TSA software version 0.9.5.10 beta. We set several effect sizes of clinical relevance and made conclusions according to three conditions: required information size, whether the monitoring boundary was crossed, and whether the futility boundary was crossed [75–77].

Publication bias was assessed using funnel plots. In general, a two-sided *P* value < 0.05 is considered statistically significant. However, in this meta-analysis, alpha error correction was considered using the Bonferroni adjustment. Seven outcomes (one primary and six secondary outcomes) were evaluated; therefore, the significance level was set at 0.0071 (0.05/6). Similarly, the significance level was set at 0.0042 (0.05/12), because a total of 12 subgroup analyses were conducted. The risk-of-bias plot and funnel plot were generated using Review Manager (RevMan) version 5.3 software (Nordic Cochrane Centre, The Cochrane Collaboration, 2014). This meta-analysis as well as the random and mixed effect models was conducted using Comprehensive Meta-Analysis version 3 software (Biostat, Englewood, NJ, 2013).

## Results

### Literature search

**Fig. 1:**
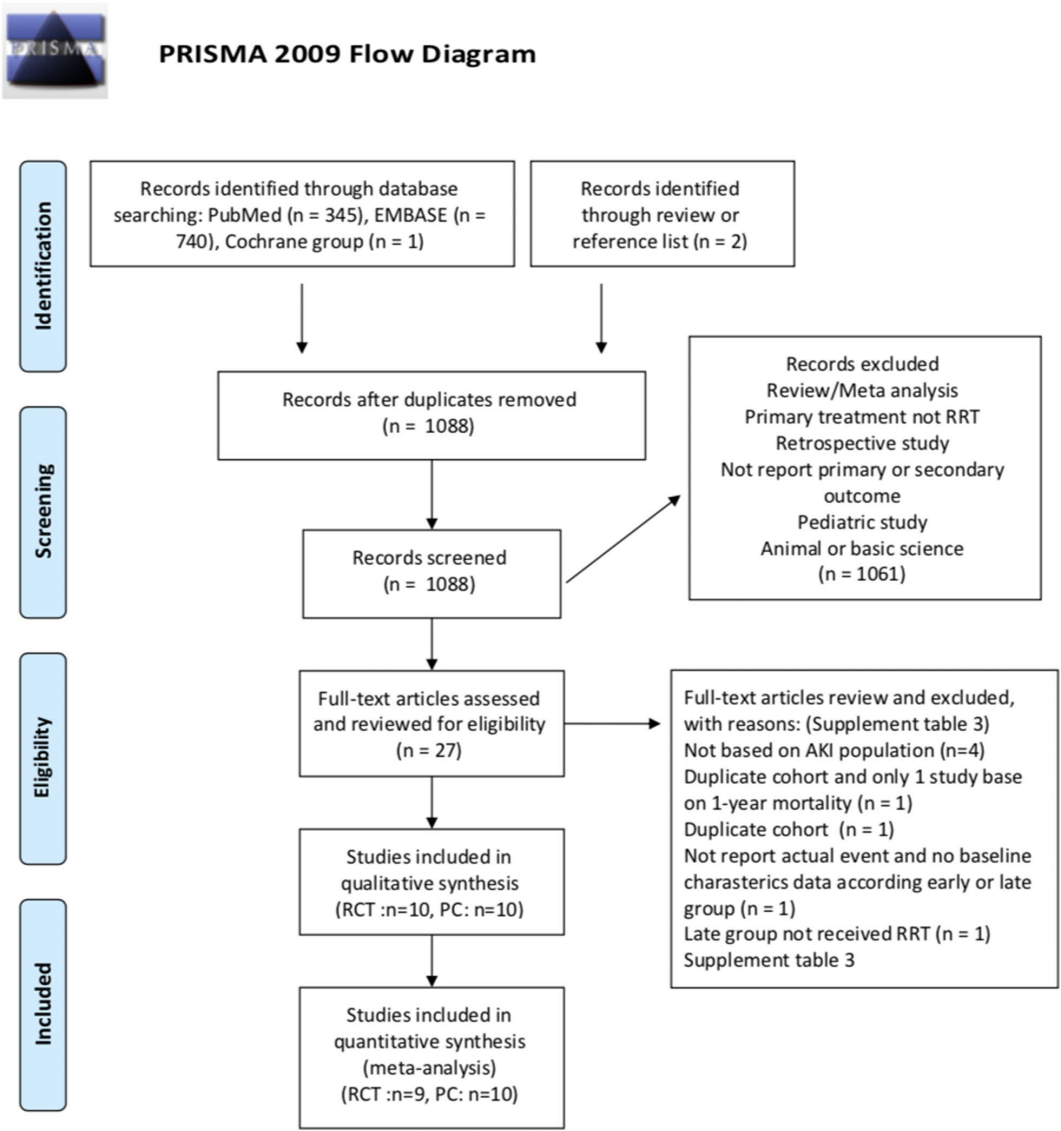
PRISMA flowchart of study inclusion

In the initial search, we retrieved articles using the aforementioned search strategy (Additional file 1: Table S1). After excluding duplicate articles and nonrelevant articles, the titles and abstracts of the remaining 1088 articles were screened. Twenty-seven articles were identified to be potentially relevant, and full-text articles were downloaded and assessed for eligibility. Of these 27 articles, 7 were excluded because they were not based on the AKI population [13–15, 29], RRT was not the primary treatment in the late group [30], they had a duplicate cohort with a single reported outcome that could not be used for meta-analysis [31], and no actual event numbers and no information on the baseline characteristics of the early or late RRT initiation groups were available [32] (Additional file 1: Table S5). Finally, 20 studies (9 RCTs, 10 prospective cohort studies, and 1 post hoc analysis of the AKIKI study) were included in this meta-analysis (Fig. 1).

### Study characteristics

A total of 1938 patients with AKI in 9 RCTs (excluding the post hoc analysis of the AKIKI study) were included in the meta-analysis. Our study mainly focused on RCTs; the results of 10 prospective studies are provided in Additional file 2: Document S2. Overall, 6 of the 9 included RCTs enrolled a mixed population of critically ill surgical and medical patients. Moreover, 2 of 9 RCTs enrolled surgical patients [12, 56]. One study enrolled only medical patients with septic shock [57]. The modality of RRT and the definition of AKI varied across the studies. These details are provided in Additional file 2: Document S3.

### Risk of bias

**Fig. 2:**
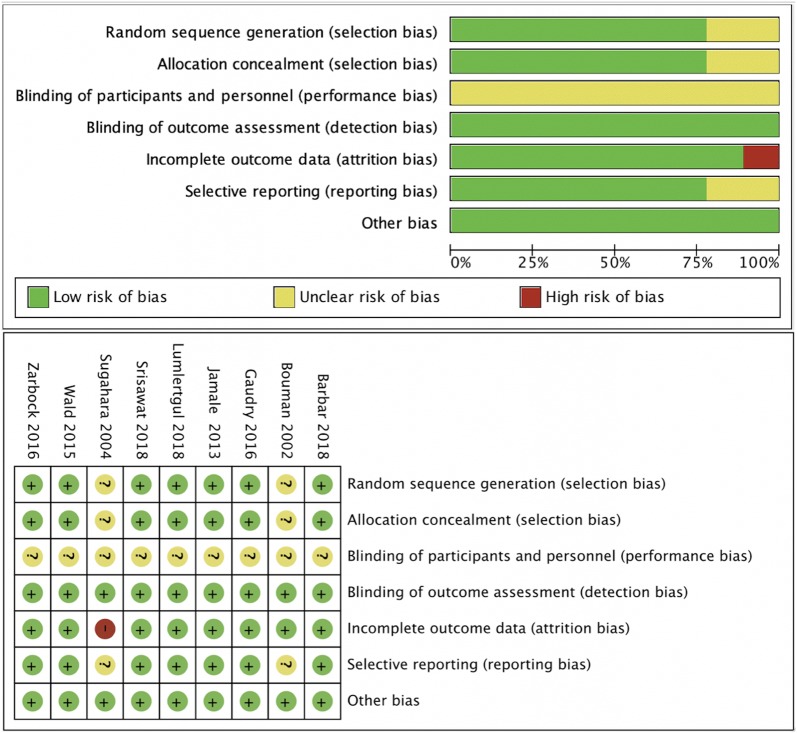
Summary of risk of bias and applicability concern

The Cochrane Collaboration’s tool for assessing the risk of bias revealed that each study had low risk or unclear risk in most domains of bias assessment. Risk-of-bias assessments of the included RCTs are summarized in Fig. 2. A detailed explanation is provided in Additional file 2: Document S4.

### Primary outcomes

**Fig. 3:**
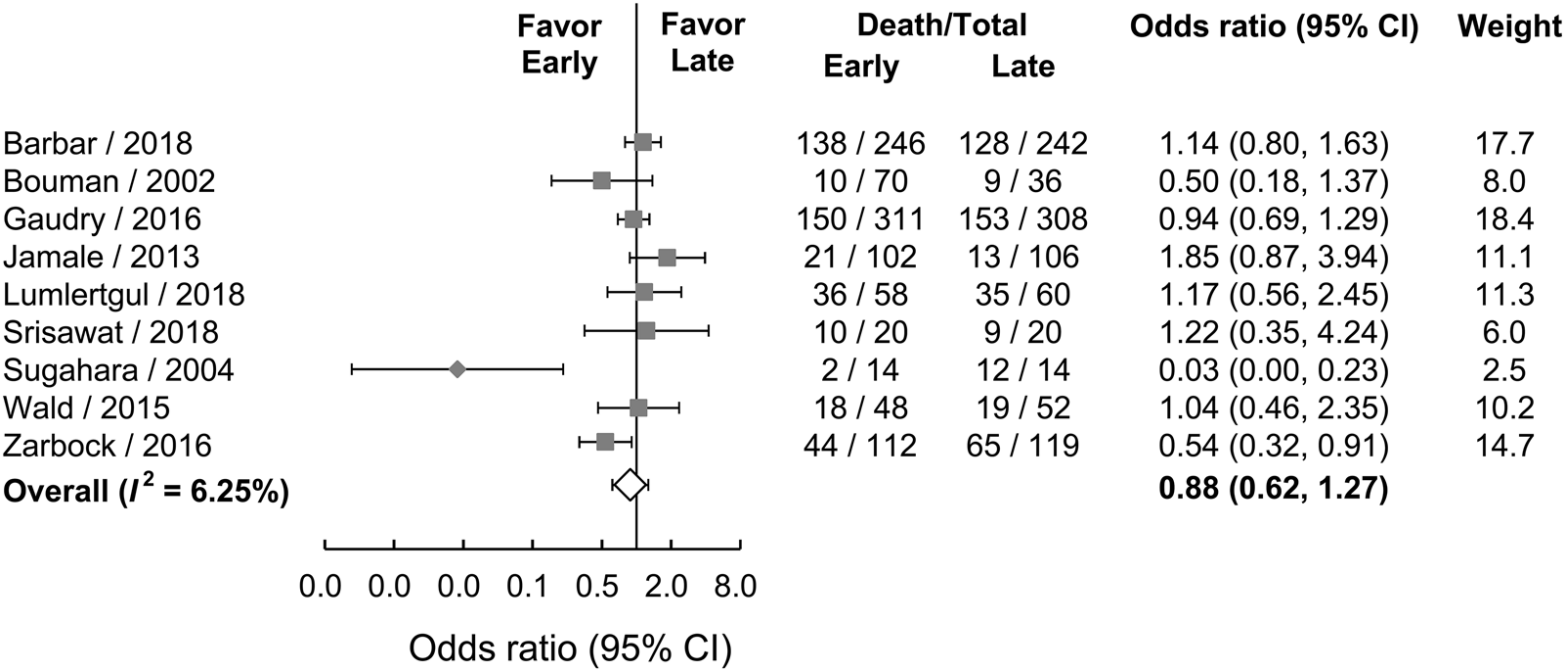
Forest plot for comparison of mortality between the early and late renal replacement therapy initiation groups in the included randomized controlled trials

Among the nine RCTs, the pooled OR for mortality in the early RRT initiation group was 0.88 (95% confidence interval [CI] 0.62–1.27) (Fig. 3). The observation period of mortality varied across studies (Additional file 1: Table S4). The results were not altered in the cumulative meta-analysis.

### Secondary outcomes

The mean difference in ICU LOS was nearly neutral: 0.09 days (95% CI − 1.01 to 1.19 days). In addition, hospital LOS was nonsignificantly different between the groups (− 1.76 days; 95% CI − 3.77 to 0.25 days). Pooled analysis showed that MV days were slightly fewer in the early RRT initiation group (−3.98 days; 95% CI − 7.81 to − 0.15 days), but this result was not statistically significant after alpha error correction. No difference was noted in RRT days between the early and late RRT initiation groups (− 2.85 days; 95% CI − 6.07 to 0.36 days). The pooled OR showed no significant difference between the groups in terms of RRT dependence (OR, 0.74; 95% CI 0.49 to 1.11) or renal recovery (OR, 1.40; 95% CI 0.75 to 2.59) (see forest plot of secondary outcomes in Additional file 3: Figure S1). In addition, the results were not altered in the cumulative meta-analysis, except for the result for MV days. The result for MV days was not significant after an outlier study [59] was excluded (data not shown).

### Subgroup analysis

We analyzed subgroups on the basis of the patient population (medical, surgical, or mixed), the use of high plasma NGAL as an enrollment criterion (yes or no), and study design (excluding or including patients with emergent need for RRT before randomization) in all nine enrolled RCTs with available outcomes. No significant modification effects were observed for three subgroup variables: mortality (included in all nine RCTs), ICU LOS (included in six RCTs: [28, 48, 55–58]), and MV days (included in six RCTs: [28, 55–58]) (Tables 1 and 2). However, all subgroup variables exerted significant modification effects on RRT days (included in six RCTs: [54–59]). The results demonstrated that RCTs that enrolled a surgical population (*P* for interaction = .001), enrolled a population with high plasma NGAL (*P* for interaction = .031 [result was nonsignificant after alpha error correction]), and did not exclude patients with the emergent need for RRT (*P* for interaction < .001) had favorable outcomes in the early RRT initiation group compared with the late RRT initiation group (Table 2). In addition, the interaction effect of RRT days subgrouped by high plasma NGAL-population was nonsignificant after an outlier study was excluded (data not shown).

**Table 1:** Subgroup analysis of mortality in the included randomized controlled trials

Outcome/subgroup	No. of studies	No. of events/no. of patients, early group	No. of events/no. of patients, late group	Pooled odds ratio (95% CI)	*I*^2^ (%)	*P* for subgroup difference
Population						0.360
Medical	1	138/246	128/242	1.14 (0.80 to 1.63)	0	
Surgical	2	46/126	77/133	0.15 (0.01 to 2.62)	85.9	
Mixed	6	245/609	238/582	1.02 (0.80 to 1.30)	0	
NGAL-based						0.969
No	5	321/743	315/706	0.85 (0.49 to 1.47)	75.1	
Yes	4	108/238	128/251	0.84 (0.54 to 1.30)	26.5	
Emergent need for RRT						0.201
Included	3	22/104	30/70	0.32 (0.06 to 1.77)	78.1	
Excluded	6	407/877	413/887	1.00 (0.75 to 1.32)	43

**Table 2:** Subgroup analysis of ICU days, MV days, and RRT days in the included randomized controlled trials

Outcome/subgroup	No. of studies	No. of patients	Pooled mean difference (95% CI)	*I*^2^ (%)	*P* for subgroup difference
ICU LOS					
Population					0.518
Medical	1	488	1.00 (− 0.91 to 2.91)	0	
Surgical	1	231	− 0.50 (− 4.65 to 3.65)	0	
Mixed	4	943	− 0.35 (− 1.77 to 1.08)	0	
NGAL-based					0.350
No	3	1213	0.30 (− 0.89 to 1.49)	0	
Yes	3	449	− 1.20 (− 4.12 to 1.72)	0	
Emergent need for RRT					0.470
Included	1	106	− 2.00 (− 7.78 to 3.78)	0	
Excluded	5	1556	0.17 (− 0.95 to 1.29)	0	
MV days					
Population					0.054
Medical	1	488	1.00 (− 1.63 to 3.63)	0	
Surgical	1	231	− 2.31 (− 4.50 to − 0.13)	0	
Mixed	4	883	− 7.14 (− 15.05 to 0.76)	89	
NGAL-based					0.101
No	3	1213	− 0.28 (− 2.01 to 1.45)	0	
Yes	3	389	− 9.44 (− 20.25 to 1.38)	92.2	
Emergent need for RRT					0.269
Included	2	146	− 13.28 (− 34.83 to 8.28)	94.6	
Excluded	4	1456	− 1.09 (− 2.78 to 0.60)	29.7	
RRT days					
Population					0.001
Medical	1	488	2.00 (1.21 to 2.79)	0	
Surgical	1	231	− 16.00 (− 28.57 to − 3.43)	0	
Mixed	4	985	− 5.19 (− 11.25 to 0.86)	93	
NGAL-based					0.031^#^
No	3	1315	1.46 (0.21 to 2.71)	60.4	
Yes	3	389	− 14.38 (− 28.74 to − 0.02)	88.1	
Emergent need for RRT					< 0.001
Included	1	40	− 24.50 (− 32.84 to − 16.16)	0	
Excluded	5	1664	0.45 (− 1.47 to 2.37)	74.5	

*LOS* length of stay, *NGAL* neutrophil gelatinase-associated lipocalin, *RRT* renal replacement therapy

^#^This result was nonsignificant at *P* < .0042 (0.05/12) if a Bonferroni adjustment was made for alpha error correction

### Sensitivity analysis

Only six [48, 54–58] of the nine RCTs fulfilled our predefined criteria for examining the safety of the watchful waiting strategy (Table 3). The pooled data of these six studies revealed no significant intergroup differences in primary and secondary outcomes (Additional file 1: Table S8A, B).

**Table 3:** Six major randomized controlled trials included in sensitivity analysis for examination of the watchful waiting strategy

Trial, year	Jamale 2013	STARRT-AKI pilot 2015	ELAIN-2016	AKIKI-2016	IDEAL-ICU 2018	The FST trial 2018
Population, *N*	Mixed, 208 (only 3 surgical)	Mixed, 100 (56% sepsis)	Surgical, 231	Mixed, 619 (80% medical)	Medical, 488 (septic shock)	Mixed, 118 (67% medical)
Inclusion criteria	AKI Urea > 70 mg/dL or creatinine > 7 mg/dL	Severe AKI (any 2 criteria): (1) 2× increase Cr (2) UOP < 6 mL/kg/12 h (3) blood NGAL ≥ 400 ng/mL)	KDIGO stage 2 and plasma NGAL > 150 ng/mL and (any one): (1) Severe sepsis (2) Catecholamine (3) Refractory fluid overload (4) Non-renal organ failure, SOFA ≥ 2	KDIGO stage 3	RIFLE-F stage and septic shock within 48 h	AKI (KDIGO) and (1) Clinical diagnosis of ATN and (2) FST nonresponsive: urine output < 200 ml for 2 h
NGAL level	NR	400 ng/mL Median: > 1300 ng/mL	> 150 ng/mL Median 490 ng/mL (early) 618 ng/mL (late)	NR	NR	≥ 150 ng/mL Median 625 ng/mL (early) 860 ng/mL (late)
Other late criteria in addition to conventional indication	Nil	Nil	AKI progression (KDIGO stage 2 to 3)	AKI non-recovery (oliguria/anuria) > 72 h)	AKI non-recovery (UOP < 1000 in diuretics naive or < 2000 with diuretics) > 48 h	Nil
Late group median from randomization to RRT (IQR), hours	NR	31.57 (22.83–59.50) Mean and SD: 51.63 h (51.95)	25.5 (18.8, 40.3) Mean and SD: 40.0 h (54.5)	57 (28–53)	51.5 (34.6–59.5)	21 (16.75–48.5)
Mortality (event/number)	21/102 vs. 13/106 (90 days)	18/48 vs. 19/52 (90 days)	44/112 vs. 65/119 (90 days)	150/311 vs. 153/308 (60 days)	138/246 vs. 128/242 (60 days)	36/58 vs. 35/60 (28 days)

*AKI* acute kidney injury; *ATN* acute tubular necrosis; *Cr* creatinine; *FST* furosemide stress test; *IQR* interquartile range; *KDIGO* Kidney Disease: Improving Global Outcomes; *NGAL* neutrophil gelatinase-associated lipocalin; *NR* not reported; *RIFLE* risk, injury, failure, loss, end-stage kidney disease; *SD* standard deviation; *UOP* urine output

### Publication bias

A funnel plot was generated to evaluate the possibility of publication bias. The results showed generally symmetrical distributions for mortality, ICU LOS, MV days, and RRT days (Additional file 4: Figure S2).

### Assessment of evidence quality and summary of findings

We evaluated the primary outcome (overall mortality) and six secondary outcomes (ICU LOS, hospital LOS, RRT dependence, renal recovery, MV days, and RRT days), and we performed quality assessment using the GRADE system. The outcomes and assessments are presented as a summary of findings in Additional file 1: Table S9.

## Discussion

In the present meta-analysis, four findings are worth summarizing. First, early RRT initiation did not reduce all-cause mortality in the AKI population. Second, ICU or hospital LOS, RRT duration, or renal recovery/RRT dependence was not different in the early or late RRT initiation groups, but the early RRT initiation group had fewer MV days but this result was not statistically significant after alpha error correction. Third, subgroup analysis revealed that in the surgical population and the AKI population with high plasma NGAL, early RRT initiation could reduce RRT days. Fourth, the initiation of RRT until the appearance of conventional indications in patients with AKI without conventional criteria for RRT initiation initially may not adversely affect patient outcomes.

### Previous studies

Several recent meta-analyses examined the effect of early RRT initiation on patient survival and related outcomes [5–11]. All but one meta-analysis [11] reported that the early initiation of RRT did not provide a survival advantage (Additional file 1: Table S7). However, most of these meta-analyses included trials that did not focus on the AKI population [13–15, 29]. Further discussion is included in Additional file 2: Document S5.

### Present study

Our present review did not demonstrate a statistically significant survival benefit of early RRT initiation, which is in line with the findings of most previous reviews [5–10]. Several studies that revealed positive [13] or negative [14, 29, 30] effects of early RRT initiation were not included in our meta-analysis. Early renal support has several hypothetical benefits: fluid balance maintenance, electrolyte and acid–base homeostasis, early organ support despite renal function in patients with multiple organ failure, removal of cytokines, and balance of metabolic demand in high catabolism condition (sepsis) [40]. KDIGO guidelines suggest starting RRT emergently in the event of life-threatening conditions (hyperkalemia, metabolic acidemia, pulmonary edema, and uremic complications) considered the conventional indicators for RRT initiation [26]. The 2016 Acute Disease Quality Initiative XVII International Consensus Conference suggested a more personalized consideration, and that RRT should be started when the demand exceeds the total renal capacity [41]. Only two RCTs could demonstrate a significant benefit of early RRT initiation [12, 56]. Several limitations and possible confounders might need to be considered (detailed discussion in Additional file 2: Document S6).

The present meta-analysis demonstrated a possible benefit of early RRT initiation: fewer MV days in the overall enrolled population and shorter RRT duration in subgroup analysis. However, type I error in the present study should be considered because TSA demonstrated an insufficient accrued sample size (Additional file 5: Figure S3). Further detailed discussion and results of TSA regarding the possible benefit of fewer MV days are provided in Additional file 2: Document S7.

In conventional meta-analysis, the watchful waiting strategy was found to be safe. TSA revealed that early RRT initiation was unlikely to produce a 15% or greater mortality risk reduction (Additional file 6: Figure S4). Other secondary outcomes were also examined, but an inclusive result was reported because of the relatively small sample size (Additional file 1: Table S10A, B and detailed discussion in Additional file 2: Document S8). Three of six RCTs in our sensitivity analysis used conventional criteria alone, and the others used a combination of non-renal recovery or AKI progression as the late RRT initiation criteria. In five RCTs, the time from enrollment in the study to RRT initiation varied in the late initiation group (median time ranging from 21 to 57 h) (Table 3). It is difficult to define the longest safe observation time for the watchful waiting strategy, but 1–3 days may be reasonable. We summarized different early and late RRT initiation criteria as well as the primary outcome of eight RCTs using conventional criteria as late criteria, and this information is provided in Additional file 7: Figure S5.

Although we included studies published after 2000, a possible time-dependent effect as a confounder should be considered. Compared with more recent trials, earlier studies differed in RRT prescription, AKI definition, and study design. We summarize the different RRT prescriptions in Additional file 1: Table S11. The role of biomarkers in AKI patient risk prediction and triage was noted only in recent studies and was not addressed in earlier trials. In addition, the adequate prescribed dose of RRT was more standardized after the publication of KDIGO AKI guidelines [26]. For example, in an earlier study by Bouman et al. [28], the minimal prescribed continuous venovenous hemofiltration dose was 24 L/day, which may be inadequate, as indicated by later studies and international guidelines [26, 71, 73]. Therefore, the confounding effect across time among these studies between 2002 and 2018 should be considered.

Our study has some limitations. First, significant heterogeneity is still a substantial concern, as was true in previous meta-analyses. Second, with the relatively small accrued information size as per the study by Feng et al. [7], our results should be interpreted with caution. Further information from STARRT-AKI (NCT02568722), AKIKI2 (NCT03396757), and other large-scale, well-designed RCTs (excluding patients with the emergent need for RRT before randomization, balancing underlying severity in both arms, excluding patients with severe fluid overload) should be obtained to provide a more definitive answer regarding the optimal timing of RRT initiation and optimal RRT duration. The strengths of our study are the inclusion of three recent RCTs and the fact that this study is the first subgroup analysis examining the safety of the watchful waiting strategy regarding the role of early RRT initiation in patients with AKI with high plasma NGAL.

## Conclusions

Early RRT initiation may not provide a survival advantage, but it offers the possible benefits of reduced MV and RRT days. Further well-designed studies are needed to confirm the benefit of shorter duration of MV or RRT in the surgical population and high-risk AKI population (high plasma NGAL). Our findings imply that the watchful waiting strategy chosen by nephrologists or intensivists may be a safe approach for AKI intervention.

## Supplementary information

**Additional file 1.** Additional tables.

**Additional file 2.** Additional documents.

**Additional file 3: Figure S1.** Forest plot for comparison of intensive care unit stay (A), hospital days (B), mechanical ventilator days (C), renal replacement therapy (RRT) days (D), RRT dependence (E), and renal recovery (F) between the early and late RRT initiation groups in the included randomized controlled trials.

**Additional file 4: Figure S2.** Funnel plot for evaluation of publication bias of mortality (A), renal replacement therapy days (B), mechanical ventilator days (C), intensive care unit stay (D), and hospital days (E) in the included randomized controlled trials.

**Additional file 5: Figure S3.** Trial Sequential Analysis of mechanical ventilation days in the included randomized controlled trials.

**Additional file 6: Figure S4.** Trial Sequential Analysis of mortality with an estimated relative risk reduction of 15% between the early and late renal replacement therapy initiation groups in the six randomized controlled trials with the watchful waiting strategy.

**Additional file 7: Figure S5.** Summary of early and late renal replacement therapy initiation criteria and primary outcome of the eight randomized controlled trials using conventional criteria as late criteria.

## Data Availability

Not applicable.

## Abbreviations

AKI: Acute kidney injury
ATN: Acute tubular necrosis
Cr: Creatinine
ICU: Intensive care unit
KDIGO: Kidney Disease Improving Global Outcomes
NGAL: Neutrophil gelatinase-associated lipocalin
LOS: Length of stay
MV: Mechanical ventilation
RCT: Randomized controlled trial
RIFLE: Risk, injury, failure, loss of kidney function, and end-stage kidney disease
RRT: Renal replacement therapy
UOP: Urine output

## References

[1] Carlson N, Hommel K, Olesen JB, Soja AM, Vilsboll T, Kamper AL (2016). Trends in one-year outcomes of dialysis-requiring acute kidney injury in Denmark 2005–2012: a population-based nationwide study. PLoS ONE.

[2] Susantitaphong P, Cruz DN, Cerda J, Abulfaraj M, Alqahtani F, Koulouridis I (2013). World incidence of AKI: a meta-analysis. Clin J Am Soc Nephrol.

[3] Clec’h C, Gonzalez F, Lautrette A, Nguile-Makao M, Garrouste-Orgeas M, Jamali S (2011). Multiple-center evaluation of mortality associated with acute kidney injury in critically ill patients: a competing risks analysis. Crit Care.

[4] Hoste EA, Bagshaw SM, Bellomo R, Cely CM, Colman R, Cruz DN (2015). Epidemiology of acute kidney injury in critically ill patients: the multinational AKI-EPI study. Intensive Care Med.

[5] Fayad AII, Buamscha DG, Ciapponi A (2018). Timing of renal replacement therapy initiation for acute kidney injury. Cochrane Database Syst Rev.

[6] Cui J, Tang D, Chen Z, Liu G (2018). Impact of early versus late initiation of renal replacement therapy in patients with cardiac surgery-associated acute kidney injury: meta-analysis with trial sequential analysis of randomized controlled trials. Biomed Res Int.

[7] Feng YM, Yang Y, Han XL, Zhang F, Wan D, Guo R (2017). The effect of early versus late initiation of renal replacement therapy in patients with acute kidney injury: a meta-analysis with trial sequential analysis of randomized controlled trials. PLoS ONE.

[8] Lai TS, Shiao CC, Wang JJ, Huang CT, Wu PC, Chueh E (2017). Earlier versus later initiation of renal replacement therapy among critically ill patients with acute kidney injury: a systematic review and meta-analysis of randomized controlled trials. Ann Intensive Care..

[9] Yang XM, Tu GW, Zheng JL, Shen B, Ma GG, Hao GW (2017). A comparison of early versus late initiation of renal replacement therapy for acute kidney injury in critically ill patients: an updated systematic review and meta-analysis of randomized controlled trials. BMC Nephrol..

[10] Wierstra BT, Kadri S, Alomar S, Burbano X, Barrisford GW, Kao RL (2016). The impact of “early” versus “late” initiation of renal replacement therapy in critical care patients with acute kidney injury: a systematic review and evidence synthesis. Crit Care.

[11] Liu Y, Davari-Farid S, Arora P, Porhomayon J, Nader ND (2014). Early versus late initiation of renal replacement therapy in critically ill patients with acute kidney injury after cardiac surgery: a systematic review and meta-analysis. J Cardiothorac Vasc Anesth.

[12] Sugahara S, Suzuki H (2004). Early start on continuous hemodialysis therapy improves survival rate in patients with acute renal failure following coronary bypass surgery. Hemodial Int..

[13] Durmaz I, Yagdi T, Calkavur T, Mahmudov R, Apaydin AZ, Posacioglu H (2003). Prophylactic dialysis in patients with renal dysfunction undergoing on-pump coronary artery bypass surgery. Ann Thorac Surg.

[14] Payen D, Mateo J, Cavaillon JM, Fraisse F, Floriot C, Vicaut E (2009). Impact of continuous venovenous hemofiltration on organ failure during the early phase of severe sepsis: a randomized controlled trial. Crit Care Med.

[15] Combes A, Brechot N, Amour J, Cozic N, Lebreton G, Guidon C (2015). Early high-volume hemofiltration versus standard care for post-cardiac surgery shock. The HEROICS Study. Am J Respir Crit Care Med.

[16] Klein SJ, Brandtner AK, Lehner GF, Ulmer H, Bagshaw SM, Wiedermann CJ (2018). Biomarkers for prediction of renal replacement therapy in acute kidney injury: a systematic review and meta-analysis. Intensive Care Med.

[17] Siew ED, Ware LB, Gebretsadik T, Shintani A, Moons KG, Wickersham N (2009). Urine neutrophil gelatinase-associated lipocalin moderately predicts acute kidney injury in critically ill adults. J Am Soc Nephrol.

[18] Seibert FS, Pagonas N, Arndt R, Heller F, Dragun D, Persson P (2013). Calprotectin and neutrophil gelatinase-associated lipocalin in the differentiation of pre-renal and intrinsic acute kidney injury. Acta Physiol.

[19] Zhang A, Cai Y, Wang PF, Qu JN, Luo ZC, Chen XD (2016). Diagnosis and prognosis of neutrophil gelatinase-associated lipocalin for acute kidney injury with sepsis: a systematic review and meta-analysis. Crit Care.

[20] Chang CH, Yang CH, Yang HY, Chen TH, Lin CY, Chang SW (2015). Urinary biomarkers improve the diagnosis of intrinsic acute kidney injury in coronary care units. Medicine.

[21] Fan PC, Chang CH, Chen YC (2018). Biomarkers for acute cardiorenal syndrome. Nephrology.

[22] Koyner JL, Davison DL, Brasha-Mitchell E, Chalikonda DM, Arthur JM, Shaw AD (2015). Furosemide stress test and biomarkers for the prediction of AKI severity. J Am Soc Nephrol.

[23] Meersch M, Schmidt C, Hoffmeier A, Van Aken H, Wempe C, Gerss J (2017). Prevention of cardiac surgery-associated AKI by implementing the KDIGO guidelines in high risk patients identified by biomarkers: the PrevAKI randomized controlled trial. Intensive Care Med.

[24] Bellomo R, Ronco C, Kellum JA, Mehta RL, Palevsky P, Acute Dialysis Quality Initiative workgroup (2004). Acute renal failure—definition, outcome measures, animal models, fluid therapy and information technology needs: the Second International Consensus Conference of the Acute Dialysis Quality Initiative (ADQI) Group. Crit Care.

[25] Mehta RL, Kellum JA, Shah SV, Molitoris BA, Ronco C, Warnock DG (2007). Acute Kidney Injury Network: report of an initiative to improve outcomes in acute kidney injury. Crit Care.

[26] Kellum JA, Lameire N, Aspelin P, Barsoum RS, Burdmann EA, Goldstein SL, Herzog CA, Joannidis M, Kribben A, Levey AS, MacLeod AM (2012). Kidney disease: improving global outcomes (KDIGO) acute kidney injury work group. KDIGO clinical practice guideline for acute kidney injury. Kidney Int Suppl.

[27] Higgins JP, Altman DG, Gotzsche PC, Juni P, Moher D, Oxman AD (2011). The Cochrane Collaboration’s tool for assessing risk of bias in randomised trials. BMJ.

[28] Bouman CS, Oudemans-Van Straaten HM, Tijssen JG, Zandstra DF, Kesecioglu J (2002). Effects of early high-volume continuous venovenous hemofiltration on survival and recovery of renal function in intensive care patients with acute renal failure: a prospective, randomized trial. Crit Care Med.

[29] Christiansen S, Christensen S, Pedersen L, Gammelager H, Layton JB, Brookhart MA (2017). Timing of renal replacement therapy and long-term risk of chronic kidney disease and death in intensive care patients with acute kidney injury. Crit Care.

[30] Elseviers MM, Lins RL, Van der Niepen P, Hoste E, Malbrain ML, Damas P (2010). Renal replacement therapy is an independent risk factor for mortality in critically ill patients with acute kidney injury. Crit Care.

[31] Meersch M, Kullmar M, Schmidt C, Gerss J, Weinhage T, Margraf A (2018). Long-term clinical outcomes after early initiation of RRT in critically ill patients with AKI. J Am Soc Nephrol.

[32] Dlamini TAL, Heering PJ, Chivese T, Rayner B (2017). A prospective study of the demographics, management and outcome of patients with acute kidney injury in Cape Town, South Africa. PLoS ONE.

[33] Gaudry S, Hajage D, Schortgen F, Martin-Lefevre L, Verney C, Pons B (2018). Timing of renal support and outcome of septic shock and acute respiratory distress syndrome. A post hoc analysis of the AKIKI randomized clinical trial. Am J Respir Crit Care Med.

[34] Iyem H, Tavli M, Akcicek F, Buket S (2009). Importance of early dialysis for acute renal failure after an open-heart surgery. Hemodial Int..

[35] Crescenzi G, Torracca L, Pierri MD, Rosica C, Munch C, Capestro F (2015). ‘Early’ and ‘late’ timing for renal replacement therapy in acute kidney injury after cardiac surgery: a prospective, interventional, controlled, single-centre trial. Interact Cardiovasc Thorac Surg..

[36] Karvellas CJ, Farhat MR, Sajjad I, Mogensen SS, Leung AA, Wald R (2011). A comparison of early versus late initiation of renal replacement therapy in critically ill patients with acute kidney injury: a systematic review and meta-analysis. Crit Care.

[37] Seabra VF, Balk EM, Liangos O, Sosa MA, Cendoroglo M, Jaber BL (2008). Timing of renal replacement therapy initiation in acute renal failure: a meta-analysis. Am J Kidney Dis.

[38] Schneider AG, Bellomo R, Bagshaw SM, Glassford NJ, Lo S, Jun M (2013). Choice of renal replacement therapy modality and dialysis dependence after acute kidney injury: a systematic review and meta-analysis. Intensive Care Med.

[39] Forni LG, Darmon M, Ostermann M, Oudemans-van Straaten HM, Pettila V, Prowle JR (2017). Renal recovery after acute kidney injury. Intensive Care Med.

[40] Zarbock A, Mehta RL (2019). Timing of kidney replacement therapy in acute kidney injury. Clin J Am Soc Nephrol.

[41] Ostermann M, Joannidis M, Pani A, Floris M, De Rosa S, Kellum JA (2016). Patient selection and timing of continuous renal replacement therapy. Blood Purif.

[42] Shiao CC, Huang TM, Spapen HD, Honore PM, Wu VC (2017). Optimal timing of renal replacement therapy initiation in acute kidney injury: the elephant felt by the blindmen?. Crit Care.

[43] Gaudry S, Quenot JP, Hertig A, Barbar SD, Hajage D, Ricard JD (2019). Timing of renal replacement therapy for severe acute kidney injury in critically ill patients. Am J Respir Crit Care Med.

[44] Barbar SD, Gaudry S, Dreyfuss D, Quenot JP (2018). Renal replacement therapy: time to give up on early initiation? Yes. Anaesth Crit Care Pain Med..

[45] Payen D, de Pont AC, Sakr Y, Spies C, Reinhart K, Vincent JL (2008). A positive fluid balance is associated with a worse outcome in patients with acute renal failure. Crit Care.

[46] Kim IY, Kim JH, Lee DW, Lee SB, Rhee H, Seong EY (2017). Fluid overload and survival in critically ill patients with acute kidney injury receiving continuous renal replacement therapy. PLoS ONE.

[47] Bouchard J, Soroko SB, Chertow GM, Himmelfarb J, Ikizler TA, Paganini EP (2009). Fluid accumulation, survival and recovery of kidney function in critically ill patients with acute kidney injury. Kidney Int.

[48] Wald R, Adhikari NK, Smith OM, Weir MA, Pope K, Cohen A (2015). Comparison of standard and accelerated initiation of renal replacement therapy in acute kidney injury. Kidney Int.

[49] Prowle JR, Echeverri JE, Ligabo EV, Ronco C, Bellomo R (2010). Fluid balance and acute kidney injury. Nat Rev Nephrol..

[50] Lowell JA, Schifferdecker C, Driscoll DF, Benotti PN, Bistrian BR (1990). Postoperative fluid overload: not a benign problem. Crit Care Med.

[51] Bagshaw SM, Uchino S, Bellomo R, Morimatsu H, Morgera S, Schetz M (2007). Septic acute kidney injury in critically ill patients: clinical characteristics and outcomes. Clin J Am Soc Nephrol.

[52] Jaber S, Paugam C, Futier E, Lefrant JY, Lasocki S, Lescot T (2018). Sodium bicarbonate therapy for patients with severe metabolic acidaemia in the intensive care unit (BICAR-ICU): a multicentre, open-label, randomised controlled, phase 3 trial. Lancet.

[53] Sabater JPX, Albertos R, Gutierrez D, Labad X (2009). Acute renal failure in septic shock: should we consider different continuous renal replacement therapies on each RIFLE score stage?. Intensive Care Med.

[54] Jamale TE, Hase NK, Kulkarni M, Pradeep KJ, Keskar V, Jawale S (2013). Earlier-start versus usual-start dialysis in patients with community-acquired acute kidney injury: a randomized controlled trial. Am J Kidney Dis.

[55] Gaudry S, Hajage D, Schortgen F, Martin-Lefevre L, Pons B, Boulet E (2016). Initiation strategies for renal-replacement therapy in the intensive care unit. N Engl J Med.

[56] Zarbock A, Kellum JA, Schmidt C, Van Aken H, Wempe C, Pavenstadt H (2016). Effect of early vs delayed initiation of renal replacement therapy on mortality in critically ill patients with acute kidney injury: the ELAIN randomized clinical trial. JAMA.

[57] Barbar SD, Clere-Jehl R, Bourredjem A, Hernu R, Montini F, Bruyere R (2018). Timing of renal-replacement therapy in patients with acute kidney injury and sepsis. N Engl J Med.

[58] Lumlertgul N, Peerapornratana S, Trakarnvanich T, Pongsittisak W, Surasit K, Chuasuwan A (2018). Early versus standard initiation of renal replacement therapy in furosemide stress test non-responsive acute kidney injury patients (the FST trial). Crit Care.

[59] Srisawat N, Laoveeravat P, Limphunudom P, Lumlertgul N, Peerapornratana S, Tiranathanagul K (2018). The effect of early renal replacement therapy guided by plasma neutrophil gelatinase associated lipocalin on outcome of acute kidney injury: a feasibility study. J Crit Care.

[60] Liu KD, Himmelfarb J, Paganini E, Ikizler TA, Soroko SH, Mehta RL (2006). Timing of initiation of dialysis in critically ill patients with acute kidney injury. Clin J Am Soc Nephrol.

[61] Bagshaw SM, Uchino S, Bellomo R, Morimatsu H, Morgera S, Schetz M (2009). Timing of renal replacement therapy and clinical outcomes in critically ill patients with severe acute kidney injury. J Crit Care.

[62] Shiao CC, Wu VC, Li WY, Lin YF, Hu FC, Young GH (2009). Late initiation of renal replacement therapy is associated with worse outcomes in acute kidney injury after major abdominal surgery. Crit Care.

[63] Jun M, Bellomo R, Cass A, Gallagher M, Lo S, Lee J (2014). Timing of renal replacement therapy and patient outcomes in the randomized evaluation of normal versus augmented level of replacement therapy study. Crit Care Med.

[64] Vaara ST, Reinikainen M, Wald R, Bagshaw SM, Pettila V, Group FS (2014). Timing of RRT based on the presence of conventional indications. Clin J Am Soc Nephrol.

[65] Lim CC, Tan CS, Kaushik M, Tan HK (2015). Initiating acute dialysis at earlier Acute Kidney Injury Network stage in critically ill patients without traditional indications does not improve outcome: a prospective cohort study. Nephrology.

[66] Park JY, An JN, Jhee JH, Kim DK, Oh HJ, Kim S (2016). Early initiation of continuous renal replacement therapy improves survival of elderly patients with acute kidney injury: a multicenter prospective cohort study. Crit Care.

[67] Cochrane Handbook for Systematic Reviews of Interventions version 5.1 online. https://training.cochrane.org/handbook. Accessed 05 Mar 2019.

[68] The Newcastle-Ottawa Scale (NOS) for assessing the quality of nonrandomised studies in meta-analyses, http://www.ohri.ca/programs/clinical_epidemiology/oxford.asp. Accessed 05 Mar 2019.

[69] GRADEpro GDT: GRADEpro Guideline Development Tool [Software], McMaster University, 2015, developed by Evidence Prime, Inc., https://gradepro.org. Accessed 05 Mar 2019.

[70] Cochrane Handbook for Systematic Reviews of Interventions, https://handbook-5-1.cochrane.org/chapter_7/7_7_3_5_mediansand_interquartile_ranges.htm. Accessed 05 Mar 2019.

[71] Pursnani ML, Hazra DK, Singh B, Pandey DN (1997). Early haemodialysis in acute tubular necrosis. J Assoc Physicians India.

[72] Vinsonneau C, Allain-Launay E, Blayau C, Darmon M, Ducheyron D, Gaillot T (2015). Renal replacement therapy in adult and pediatric intensive care: recommendations by an expert panel from the French Intensive Care Society (SRLF) with the French Society of Anesthesia Intensive Care (SFAR) French Group for Pediatric Intensive Care Emergencies (GFRUP) the French Dialysis Society (SFD). Ann Intensive Care..

[73] Brochard L, Abroug F, Brenner M, Broccard AF, Danner RL, Ferrer M (2010). An official ATS/ERS/ESICM/SCCM/SRLF statement: prevention and Management of Acute Renal Failure in the ICU Patient: an international consensus conference in intensive care medicine. Am J Respir Crit Care Med.

[74] Bagshaw SM, Wald R (2017). Strategies for the optimal timing to start renal replacement therapy in critically ill patients with acute kidney injury. Kidney Int.

[75] Wetterslev J, Thorlund K, Brok J, Gluud C (2008). Trial sequential analysis may establish when firm evidence is reached in cumulative meta-analysis. J Clin Epidemiol.

[76] Roshanov PS, Dennis BB, Pasic N, Garg AX, Walsh M (2017). When is a meta-analysis conclusive? A guide to Trial Sequential Analysis with an example of remote ischemic preconditioning for renoprotection in patients undergoing cardiac surgery. Nephrol Dial Transplant.

[77] Thorlund K, Devereaux PJ, Wetterslev J, Guyatt G, Ioannidis JPA, Thabane L (2008). Can trial sequential monitoring boundaries reduce spurious inferences from meta-analyses?. Int J Epidemiol.

