# Supplementary material for: Comparison between watchful waiting strategy and early initiation of renal replacement therapy in the critically ill acute kidney injury population: an updated systematic review and meta-analysis
Source: Ann Intensive Care. 2020 Mar 3;10:30. doi: 10.1186/s13613-020-0641-5 (PMC7054512; doi:10.1186/s13613-020-0641-5)
Supplement: Supplementary file 1 — Additional file 1. Additional tables. [file 13613_2020_641_MOESM1_ESM.docx]

**Table S1. Search strategy terms and results**

| PubMed searched in March 2019 | | |
| --- | --- | --- |
| 1 | “Acute kidney injury” OR “acute kidney failure” OR “acute renal failure” | 58,475 |
| 2 | Renal replacement therapy OR dialysis OR hemodialysis OR Continuous renal replacement therapy OR hemofiltration | 276,890 |
| 3 | Time OR early OR late OR time to treatment OR accelerate OR accelerated | 5,316,230 |
| 4 | Combine 1 AND 2 AND 3 | 4338 |
| 5 | Filters: Human | 3810 |
| 6 | Filters: English | 3257 |
| 7 | Filters: Adult (19+ y old) | 2166 |
| 8 | Filters: Clinical trial OR review OR meta-analysis | 345 |

| Embase searched in March 2019 | | |
| --- | --- | --- |
| 1 | “Acute kidney injury” OR “acute kidney failure” OR “acute renal failure” | 59,508 |
| 2 | Renal replacement therapy OR dialysis OR hemodialysis OR Continuous renal replacement therapy OR hemofiltration | 200,750 |
| 3 | Time OR early OR late OR time to treatment OR accelerate OR accelerated | 5,706,323 |
| 4 | Combine 1 AND 2 AND 3 AND 2 | 4934 |
| 6 | Filters: Controlled study, prospective study, cohort analysis, RCT | 1554 |
| 7 | Filters: Adult AND aged, 2000–2019 | 1106 |
| 7 | Filters: Publication type – article, human | 740 |

**Table S2. Summary of randomized controlled trials included in this meta-analysis**

| Author/year | Country/period | Study population | Guideline-based AKI criteria | Inclusion criteria | Exclusion criteria | Patient numbers  Total/early/late |
| --- | --- | --- | --- | --- | --- | --- |
| Bouman/2002 [28] | Netherlands/1998–2000 | Mixed | No | UOP < 30 mL/h for 6 h, CCr < 20 mL/min (3-h urine data), mechanical ventilation | Baseline eGFR < 30 mL/min (CG formula), ARF etiology (renal artery occlusion, glomerulonephritis, TIN, postrenal obstruction) , child C cirrhosis, AIDS, nonwitness cardiac arrest with CGS < 5, AIDS with low CD4 count, hematologic malignancy with neutropenia | 106/70/36 |
| Sugahara/2004 [12] | Japan/1995–1997 | Surgical (CABG) | No | UOP < 30 mL/h, Cr increase >0.5/dL/d | Pregnancy, bilirubin >5 mg/dL, mental disorder, cancer, proteinuria >2 g/d, baseline serum Cr >1.4 mg/dL | 28/14/14 |
| Jamale/2013 [54] | India/2011–2012 | Mixed | No | Urea >70 mg/dL or Creatinine >7 mg/dL | Life-threatening uremic complication, chronic dialysis, in recovery phase | 208/102/106 |
| Wald/2015 [48] | Canada/2012–2013 | Mixed | No | Severe AKI (defined by the presence of two of the following three criteria:  1. Twofold increase in serum creatinine from baseline  2. Urine output <6 mL/kg in preceding 12 h  3. Whole-blood NGAL ≥400 ng/mL | Urgent indications for RRT initiation (defined as serum potassium ≤5.5 mmol/L and serum bicarbonate ≥15 mmol/L); not low likelihood of volume-responsive AKI (defined as central venous pressure ≥8 mm Hg) , PRGN, TIN, prehospital eGFR <30 mL/min, clinical suspicion of postrenal obstruction, intoxication, RRT within 2 mo | 100/48/52 |
| Gaudry/2016 [55] | France/2013–2016 | Mixed | KDIGO | KDIGO stage 3 AKI, diagnosis of ATN (ischemic or toxic), need for mechanical ventilation or catecholamine infusion | Urea >112 mg/dL, K^+^ >6 mmol/L, pH <7.15, in the context of either pure metabolic acidosis or mixed acidosis, acute pulmonary edema due to fluid overload responsible for severe hypoxemia | 619/311/308 |
| Zarbock/2016 [56] | Germany/2013–2015 | Surgical (cardiac + surgical) | KDIGO | KDIGO stage 2 AKI despite optimal volume status, plasma NGAL >150 ng/mL, at least 1 of the following conditions: severe sepsis, use of vasopressors or catecholamines (norepinephrine or epinephrine >0.1 μg/kg/min), refractory fluid overload (worsening pulmonary edema, PaO_2_/FiO_2_ < 300 mm Hg or fluid balance >10% of body weight), development or progression of nonrenal organ dysfunction (Sequential Organ Failure Assessment [SOFA] score ≥2) | Preexisting chronic kidney disease (eGFR <30 mL/min), previous renal replacement therapy, AKI caused by permanent occlusion or surgical lesion of the renal artery, glomerulonephritis, interstitial nephritis | 231/112/119 |
| Barbar/2018 [57] | France/2012–2016 | Medical (septic shock) | RIFLE | ICU + early phase of septic shock (within 48 h after start of vasopressor therapy), RIFLE failure stage | Chronic RRT, obstructive etiology of AKI, need for emergency RRT (K^+^ >6.5 mEq/dL, metabolic acidosis with pH < 7.15, fluid overload refractory to diuretics + pulmonary edema), already receiving RRT, pregnancy, moribund with expected death within 24 h, survival to 28 d unlikely | 488/246/242 |
| Lumlertgul/2018 [58] | Thailand/2016–2017 | Mixed | KDIGO | Clinical diagnosis of ATN (granular or epithelial cast, fractional excretion of sodium ≥1%, fractional excretion of urea ≥50%, plasma NGAL ≥150 ng/mL), well resuscitated and euvolemic, neither an emergent indication nor a contraindication to RRT, FST nonresponsive: urine output <200 mL for 2 h | Baseline Cr ≥2 mg/dL (male) or ≥1.5 mg/dL (female)  History of renal allograft  Known pregnancy  Allergy or known sensitivity to loop diuretics  Moribund patients with expected death within 24 h or whose survival to 28 d was unlikely, owing to an uncontrollable comorbidity (e.g., end-stage liver or heart disease, untreatable malignancy)  Patients with advanced directives who expressed the desire not to be resuscitated  Prior treatment with RRT within 30 d  Serum albumin <2 g/dL  Patients receiving extracorporeal membrane oxygenation or circulatory assistance | 118/58/60 |
| Srisawat/2018 [59] | Thailand/2012–2014 | Mixed | RIFLE | Acute kidney injury by RIFLE criteria, plasma NGAL level ≥400 ng/mL | Life expectancy < 24 h, ESRD, baseline serum creatinine >2 mg/dL in males or >1.5 mg/dL in females, previous kidney transplant, and pregnancy | 40/20/20 |
| Total |  |  |  |  |  | 1938/981/957 |

Abbreviations: AKI: acute kidney injury, ATN: acute tubular necrosis, ARDS: acute respiratory distress syndrome, CABG: coronary artery bypass surgery, Cr: creatinine, CCr: creatinine clearance, ESRD: end-stage renal disease, KDIGO: Kidney Disease: Improving Global Outcomes, NGAL: neutrophil gelatinase–associated lipocalin, RIFLE: risk, injury, failure, loss, end-stage kidney disease , RPGN: rapid progression glomerulonephritis, RRT: renal replacement therapy, TIN: tubulointerstitial nephritis, UOP: urine output

**Table S2. Summary of randomized controlled trials included in this meta-analysis: definition of early and late criteria and outcome (continued)**

| First author/year | Early criteria | Late criteria | RRT  modality | High plasma NGAL as enrollment criterion | Conventional criteria as late criteria | Exclusion of emergent need for RRT | Outcome |
| --- | --- | --- | --- | --- | --- | --- | --- |
| Bouman/2002 | Time <12 h after inclusion | Conventional criteria (urea >40 mmol/L, K^+^ >6.5 mEq/L, severe pulmonary edema) | HF | N | Y | N | Mortality (30 d): N  RRT times: NR  MV times: N  ICU LOS: N  Hospital LOS: N |
| Sugahara/2004 | UOP < 30 mL/h for 3 h or urine output <750 mL/d | UOP < 20 mL/h for 2 h or urine output <500 mL/d | CVVHD | N | N (by UOP) | N | Mortality (14 d): E  RRT times: NR  MV times: NR  ICU LOS: NR  Hospital LOS: NR |
| Jamale/2013 | Urea >70 mg/dL or Creatinine >7 mg/dL | Conventional criteria (hyperkalemia, acidosis, fluid overload, uremic nausea/vomiting) | iHD | N | Y | Y | Mortality (30 d): N  RRT times: L  MV times: NR  ICU LOS: NR  Hospital LOS: NR |
| Wald/2015 | RRT within 12 h after inclusion | Serum K^+^ >6.0 mmol/L, serum bicarbonate <10 mmol/L, or PaO_2_/FiO_2_ <200 with infiltrates on chest radiograph compatible with pulmonary edema | iHD/CRRT/SLED | Y | Y | Y | Mortality (30 d): N  Mortality (90 d): N  RRT times: NR  MV times: NR  ICU LOS: N  Hospital LOS: N |
| Gaudry/2016 | RRT within 6 h after diagnosis of KDIGO stage 3 AKI | Urea >112 mg/dL, K^+^ >6 mmol/L, pH <7.15 , acute pulmonary edema, oliguria/anuria for 72 h | iHD/CRRT | N | Y | Y | Mortality (30 d): N  Mortality (60 d): N  RRT times: L (free)  MV times: N  ICU LOS: N  Hospital LOS: NR |
| Zarbock/2016 | RRT within 8 h after diagnosis of KDIGO stage 2 AKI | RRT within 12 h after diagnosis of KDIGO stage 3 AKI or absolute indication for RRT (urea serum levels >100 mg/dL, serum K^+^ levels >6 mmol/L, and/or ECG abnormalities); magnesium serum levels >4 mmol/L; urine production <200 mL/12 h or anuria (without diuretics, according to the KDIGO recommendations); and organ edema in the presence of AKI resistant to diuretic treatment (one attempt with loop diuretics prior to randomization) |  | Y | Y (also KDIGO stage 3 criteria) | Y | Mortality (30 d): N  Mortality (90 d): E  RRT times: E  MV times: E  ICU LOS: N  Hospital LOS: E |
| Barbar/2018 | RRT within 12 h after diagnosis of RIFLE failure stage AKI | Emergent need for RRT (K^+^ ≥6.5 mmol/L with characteristic electrocardiographic changes, metabolic acidosis [pH < 7.15]), defined as a base deficit >5 mEq/L or HCO_3_^−^ <18 mEq/L, pulmonary edema) or 48 h after diagnosis of AKI (in patients without spontaneous renal recovery) | iHD/CVVHD/CVVH/SLED | N | Y (also 48 h after diagnosis of AKI) | Y | Mortality (30 d): N  Mortality (90 d): N  Mortality (180 d): N  RRT times: L  MV times: N  ICU LOS: N  Hospital LOS: N |
| Lumlertgul/2018 | RRT within 6 h after randomization | BUN ≥100 mg/dL, serum K^+^ >6 mmol/L, serum HCO_3_^−^ <12 mmol/L or pH <7.15, PaO_2_/FiO_2_ ratio <200, or chest radiograph compatible with pulmonary edema | CVVH/PIRRT/iHD | Y | Y | Y | Mortality (30 d): N  RRT times: N  MV times: N  ICU LOS: N  Hospital LOS: N |
| Srisawat/2018 | RRT within 12 h after randomization | Refractory severe acidosis (pH <7.2 or HCO_3_^−^ <15 mEq/L), severe peripheral edema, pulmonary edema, no response to diuretics, refractory hyperkalemia (K^+^ >6.2 mEq/L or the presence of electrocardiogram change: tall T wave, absent P wave, or wide QRS wave), anuria or oliguria, or high BUN >60 mg/dL | CVVH | Y | Y | Y | Mortality (30 d): N  RRT times: NR  MV times: E  ICU LOS: N  Hospital LOS: N |

RRT times also included RRT-free days during follow-up.

MV times also included MV-free days during follow-up.

In-hospital mortality considered as 30-d mortality.

Abbreviations: ARF: Acute renal failure, Cr: creatinine, CRRT: continuous renal replacement therapy, CVVH: continuous venovenous hemofiltration, CVVHD: continuous venovenous hemodialysis, E: favor early, HF: hemofiltration, iHD: intermittent hemodialysis, L: favor late, MV: mechanical ventilation, NR: not reported, NGAL: neutrophil gelatinase–associated lipocalin, NS: nonsignificant difference, RPGN: rapid progression glomerulonephritis, RRT: renal replacement therapy, PIRRT: prolonged intermittent renal replacement therapy, SLED: sustained low-efficiency daily dialysis, TIN: tubulointerstitial nephritis, UOP: urine output

**Table S3. Summary of prospective cohort studies included in this meta-analysis**

| First author/year | Country/period | Study population | Guideline-based AKI criteria | RRT modality | Baseline illness severity | Inclusion criteria | Exclusion criteria |
| --- | --- | --- | --- | --- | --- | --- | --- |
| Liu/2006 [60] | USA/1999–2001 | Mixed | No | iHD/CVVH | Early worse  (more organ failure, more initial with CRRT) | AKI (increase of serum Cr ≥0.5 mg/dL and baseline serum Cr < 1.5 mg/dL or increase in serum Cr ≥ 1.0 mg/dL and baseline serum Cr 1.5–5.0 mg/dL) with ICU admission | Previous dialysis, kidney transplant, ARF due to urinary tract obstruction, and hypovolemia responsive to fluids; prisoners and pregnant patients |
| Bagshaw/2009 [61] | 23 Countries/2000–2001 | Mixed | No | iHD/CRRT | Early worse  (more cardiogenic shock, major operation, MV, and vasoactive drug) | Urea >84 mg/dL (30 mmol/L) and/or urine output <200 mL/12 h | ESRD, toxicity, RRT before ICU admission |
| Iyme/2009 [34] | Turkey/2004–2007 | Surgical | No | iHD | Equivalent | ARF < 0.5 mL/kg/h urinary output postoperatively and a 50% increase in preoperative BUN and creatinine levels | High preoperative BUN and Cr, chronic renal failure |
| Sabater/2009 [53] | Spain/NR | NR | RIFLE | CRRT | NR | NR | NR |
| Shiao/2009 [62] | Taiwan/2002–2005 | Surgical | RIFLE | iHD/CVVH | Early worse  (early more CKD, higher baseline serum Cr) | Abdominal surgery with postoperative AKI requiring RRT in ICU | Age <18 y, ICU stay <2 d, patients who started dialysis before surgery, patients who did not undergo abdominal surgery, or patients who underwent renal transplant, chronic dialysis |
| Jun/2014 [63] | Australia, New Zealand/2005–2008 | Mixed | RIFLE | CVVH | Early worse  (APACHE II score higher) | RENAL study group criteria (oliguria [urine output <100 mL in a 6-h period] that was unresponsive to fluid resuscitation, serum K^+^ >6.5 mmol/L, severe acidemia [pH <7.2], plasma urea >70 mg/dL, serum Cr >3.4 mg/dL, or presence of clinically significant organ edema), RIFLE injury group | Death is imminent (<24 h), has been treated with CRRT or other dialysis previously during the same hospital admission, on maintenance dialysis, body weight is <60 kg or >100 kg |
| Vaara/2014 [64] | Finland/2011–2012 | Mixed | AKIN | iHD/CRRT | Late worse  (SAPS II score higher) | FINNAKI study population with AKI and received RRT | 1. Preexisting ESRD requiring maintenance dialysis  2. Previous receipt of RRT while enrolled in the study  3. Organ donor  4. Patient not permanently living in Finland  5. Intermediate care patient  6. Transferred patient who had already participated in the study for 5 d (the data collection period of the study)  7. Absence of consent  8. Treated for intoxications  9. Transferred from other centers  10. Initially treated with molecular adsorbent recirculation system  11. Insufficient data for this analysis |
| Crescenzi/2015 [35] | Italy/2011–2013 | Surgical | RIFLE | CVVH | NR | All cardiac surgery–associated AKI without or without oliguria | Preoperative dialysis dependence, planned off-pump surgery, age <18 y, no signed informed consent by the patient (or proxy), and moribund patients (with expected death within 24 h) |
| Lim/2015 [65] | Singapore/2010–2013 | Mixed | AKIN | CRRT/SLED/SLED-f | Late worse  (higher SOFA score, more MV, and vasoactive drug) | Met AKIN AKI criteria | Initiated on dialysis prior to ICU admission, receiving chronic dialysis therapy |
| Park/2016 [66] | Korea/2009–2013 | Mixed | KDIGO | CRRT | Late worse  (APACHE II score higher) | Aged ≥65 y who started CRRT due to AKI | Chronic dialysis |

**Table S3. Summary of prospective cohort studies included in this meta-analysis (continued)**

| First author/year | Patient number  Total/early/late | Early criteria | Late criteria | Outcome |
| --- | --- | --- | --- | --- |
| Liu/2006 | 243/122/121 | BUN <76 mg/dL  Cutoff point by median | BUN ≥76 mg/dL | Mortality (30 d): N  RRT times: NR  MV times: NR  ICU LOS: NR  Hospital LOS: NR |
| Bagshaw/2009 | 1236/618/618 | Cr ≤3.49 mg/dL  Cutoff point by median | Cr >3.49 mg/dL | Mortality (30 d): L  RRT times: E  MV times: NR  ICU LOS: NR  Hospital LOS: N |
| Iyme/2009 | 185/95/90 | Acute renal failure + postoperation as soon as possible | 48 h after acute renal failure | Mortality (30 d): N  RRT times: NR  MV times: NR  ICU LOS: E  Hospital LOS: E |
| Sabater/2009 | 148/44/104 | RIFLE : R and I stages | RIFLE failure stage | Mortality (30 d): N  RRT times: NR  MV times: NR  ICU LOS: NR  Hospital LOS: NR |
| Shiao/2009 | 98/51/47 | RIFLE: 0-R stage | RIFLE: I and F stages | Mortality (30 d): N  RRT times: NR  MV times: NR  ICU LOS: NR  Hospital LOS: NR |
| Jun/2014 | 439/219/220 | From AKI diagnosis <17.6 h to RRT | From AKI diagnosis ≥17.6 h to RRT | Mortality (30 d): N  RRT times: NR  MV times: NR  ICU LOS: NR  Hospital LOS: NR |
| Vaara/2014 | 239/105/134 | Without conventional criteria | Conventional criteria (1) hyperkalemia (serum potassium>6 mEq/L), (2) severe acidosis (pH<7.15), (3) plasma urea >36 mmol/L (equals BUN=100.8 mg/dL), (4) oliguria or anuria (urine output<0.3 mL/kg/h for 24 h or anuria for 12 h) and (5) fluid overload with pulmonary edema | Mortality (90 d): E  RRT times: N  MV times: NR  ICU LOS: NR  Hospital LOS: NR |
| Crescenzi/2015 | 59/46/13 | Refractory oliguria with urine output <0.5 mL/kg/h for >6 h | Oliguria <0.5 mL/kg/h lasting for >12 h | Mortality (30 d): N  RRT times: NR  MV times: L  ICU LOS: NR  Hospital LOS: NR |
| Lim/2015 | 140/56/84 | AKIN stage 3 or AKIN stage 1–2 in the presence of predefined “compelling” conditions | Serum K^+^ ≥6.0 mmol/L, serum urea ≥84 mg/dL (30 mmol/L), arterial pH <7.25, serum bicarbonate <10 mmol/L, acute pulmonary edema, acute uremic encephalopathy or pericarditis | Mortality (30 d): E  RRT times: NR  MV times: NR  ICU LOS: N  Hospital LOS: N |
| Park/2016 | 482/241/241 | 6-h urine output ≥0.24 mL/kg/h | 6-h urine output <0.24 mL/kg/h | Mortality (30 d): E  (only hazard ratio)  RRT times: N  MV times: NR  ICU LOS: L  Hospital LOS: N |
| Total | 3269/1597/1672 |  |  |  |

Baseline illness severity depends on APACHE II, SOFA, and SAPS II scores; age; vasopressor; mechanical ventilator use; sepsis/shock; baseline renal function.

Abbreviations: Cr: creatinine, CRRT: continuous renal replacement therapy, CVVH: continuous venovenous hemofiltration, CVVHD: continuous venovenous hemodialysis, E: favor early, iHD: intermittent hemodialysis, L: favor late, NR: not reported, NGAL: neutrophil gelatinase–associated lipocalin, N: nonsignificant difference, RPGN: rapid progression glomerulonephritis, RRT: renal replacement therapy, PIRRT: prolonged intermittent renal replacement therapy, SLED: sustained low-efficiency daily dialysis, SLED-f: sustained low-efficiency diafiltration, TIN: tubulointerstitial nephritis, UOP: urine output

**Table S4. Detailed characteristics and outcomes of studies included in this meta-analysis**

| First author/year | Urea (mg/dL), mean | | Cr (mg/dL), mean | | Severity score, mean | | Patient number  Total/Early/Late | Mortality, event number | |
| --- | --- | --- | --- | --- | --- | --- | --- | --- | --- |
|  | Early | Late | Early | Late | Early | Late |  | Early | Late |
| **Randomized control trials** | | | | | | | | | |
| Bouman/2002 [28] | 47.8(ELV) , 45.6 (EHV) | 104.7 | NR | NR | APACHE II,  21.7(ELV), 23.5 (EHV) | APACHE II,  23.6 | 106/70/36 | 11(ELV) (28 days)  9(EHV) (28 days) | 9 (28 days) |
| Sugahara/2004 [12] | NR | NR | 2.9 | 3 | APACHE II,  19 | APACHE II,  18 | 28/14/14 | 2 (14 days) | 12 (14 days) |
| Jamale/2013 [54] | 71.7 | 100.9 | 7.4 | 10.4 | SOFA  7.6 | SOFA  8.2 | 208/102/106 | 21 (90 days) | 13 (90 days) |
| Wald/2015 [48] | 49.5 | 47.3 | 3.2 | 2.82 | SOFA  13.3 | SOFA  12.8 | 100/48/52 | 18 (90 days) | 19 (90 days) |
| Gaudry/2016 [55] | 53 | 54 | 3.25 | 3.2 | SOFA  10.9 | SOFA  10.8 | 619/311/308 | 129 (28 days) ,  150 (60 days) | 134 (28 days)  153 (60 days) |
| Zarbock/2016 [56] | 38.5 | 47.5 | 1.9 | 2.4 | APACHE II,  30.6 | APACHE II,  32.7 | 231/112/119 | 44 (90 days) | 65 (90 days) |
| Barbar/2018 [57] | 59.2 | 63.1 | 3.21 | 3.4 | SOFA  12.7 | SOFA  12.4 | 488/246/242 | 111 (28 days)  138 (90 days)  143 (180 days) | 102 (28 days)  128 (90 days)  134 (180 days) |
| Lumlertgul/2018 [58] | 42 | 51 | 2 | 2.5 | APACHE II,  24.5 | APACHE II,  21.8 | 118/58/60 | 36 (28 days) | 35 (28 days) |
| Srisawat/2018 [59] | NR | NR | 2.58 | 2.21 | NR | NR | 40/20/20 | 10 (28 days) | 9 (28 days) |
| **Prospective cohort studies** | | | | | | | | | |
| Liu/2006 [60] | 47.4 | 114.9 | 3.4 | 4.7 | NR | NR | 243/122/121 | 79 (28 days) | 71 (28 days) |
| Bagshaw/2009 [61] | 47.6 | 86.8 | NR | NR | SOFA  10.6 | SOFA  11.3 | 1236/618/618 | 330 (in hospital) | 441 (in hospital) |
| Iyme/2009  [34] | 54.6 | 68.2 | 2.1 | 2.9 | NR | NR | 185/95/90 | 6 (In hospital) | 5 (In hospital) |
| Sabater/2009 [53] | 50.4 | 72.8 | 2 | 4 | APACHE II,  24 | APACHE II,  27 | 148/44/104 | 20 (28 days) | 68 (28 days) |
| Shiao/2009  [62] | 68.8 | 81.9 | 3.3 | 3.8 | APACHE II,  18.2 | APACHE II,  20.5 | 98/51/47 | 22 (In hospital) | 35 (In hospital) |
| Jun/2014 [63] | NR | NR | NR | NR | NR | NR | 439/219/220 | 82 (28 days)  93 (90 days) | 84 (28 days)  102 (90 days) |
| Vaara/2014  [64] | 53.5 | 64.9 | 2.6 | 3.7 | SOFA  12 | SOFA  12.5 | 239/105/134 | 31 (90 days) | 65 (90 days) |
| Crescenzi/2015 [35] | NR | NR | NR | NR | NR | NR | 59/46/13 | 24 (in hoalital) | 10 (in hoalital) |
| Lim/2015  [65] | NR | NR | 2.6 | 2.8 | SOFA  7 | SOFA  11 | 140/56/84 | 36 (in hospital) | 37 (in hospital) |
| Park/2016  [66] | 49 | 43.5 | 1.8 | 2.9 | APACHE II,  29.2 | APACHE II,  29.5 | 482/241/241 | NR  (only Hazzard ratio) | NR  (only Hazzard ratio) |
| Total |  |  |  |  |  |  | 3269/1597/1672 |  |  |

**Table S4. Detailed characteristics and outcomes of studies included in this meta-analysis (continued)**

| First author/year | ICU length (days)  median | | Hospital length (days)  median | | RRT times (days)  median | | RRT free days  median | | Mechanical ventilator (days), median | | Mechanical ventilator free (days), median | |
| --- | --- | --- | --- | --- | --- | --- | --- | --- | --- | --- | --- | --- |
|  | Early | Early | Early | Late | Early | Late | Early | Late | Early | Late | Early | Late |
| Randomized control trials | | | | | | | | | | | | |
| Bouman/2002 | 13(ELV), 10(EHV) | 13.5 | 27(ELV), 27(EHV) | 35.5 | NR | NR | NR | NR | 192(ELV), 264(EHV) | 288 | NR | NR |
| Sugahara/2004 | NR | NR | NR | NR | NR | NR | NR | NR | NR | NR | NR | NR |
| Jamale/2013 | NR | NR | NR | NR | 6.63 | 4.7 | NR | NR | NR | NR | NR | NR |
| Wald/2015 | 11 | 13.5 | 29 | 31 | NR | NR | NR | NR | NR | NR | NR | NR |
| Gaudry/2016 | 13 | 13 | NR | NR | NR | NR | 17 | 19 | NR | NR | 7.0 | 0-22 |
| Zarbock/2016 | 15.5 | 16 | 33 | 43 | 9 | 25 | NR | NR | 125.5 | 181 | NR | NR |
| Barbar/2018 | 11 | 10 | 23 | 23 | 4 | 2 | 12 | 16 | NR | NR | 2.0 | 0-19 |
| Lumlertgul/2018 | 12 | 13.5 | 26 | 28.5 | NR | NR | 0 | 0 | NR | NR | 4.0 | 0-24 |
| Srisawat/2018 | NR | NR | NR | NR | NR | NR | NR | NR | NR | NR | 24.5 | 18-27 |
| Prospective trials | | | | | | | | | | | | |
| Liu/2006 | NR | NR | NR | NR | NR | NR | NR | NR | NR | NR | NR | NR |
| Bagshaw/2009 | NR | NR | 19 | 18 | 5 | 6 | NR | NR | NR | NR | NR | NR |
| Iyme/2009 | 1.92 | 3.73 | 11.1 | 17.1 | NR | NR | NR | NR | NR | NR | NR | NR |
| Sabater/2009 | 35 | 24 | NR | NR | NR | NR | NR | NR | NR | NR | NR | NR |
| Shiao/2009 | NR | NR | NR | NR | NR | NR | NR | NR | NR | NR | NR | NR |
| Jun/2014 | NR | NR | NR | NR | NR | NR | NR | NR | NR | NR | NR | NR |
| Vaara/2014 | NR | NR | NR | NR | 7 | 4 | NR | NR | NR | NR | NR | NR |
| Crescenzi/2015 | NR | NR | NR | NR | NR | NR | NR | NR | 34 | 21 | NR | NR |
| Lim/2015 | 5 | 8 | 29 | 34 | NR | NR | NR | NR | NR | NR | NR | NR |
| Park/2016 | 6.2 | 3.9 | 15.5 | 6.1 | 3.4 | 2.9 | NR | NR | NR | NR | NR | NR |

Abbreviations: Cr: creatinine, ELV: early low volume, EHV: early high volume, ICU: intensive care unit, NR: not reported, RRT: renal replacement therapy

**Table S5. Important prospective studies or randomized controlled trials excluded from this meta-analysis**

| First author/year | Country | Study population | Study design | Patient numbers  Total/early/late | Early criteria | Late criteria | Outcome | Reason for exclusion |
| --- | --- | --- | --- | --- | --- | --- | --- | --- |
| Pursnani/1997 [71] | India | Acute tubular necrosis | RCT | 18/17/35 | Dialysis before clinical deterioration | Clinical deterioration | Late group had better survival | Exclude severe AKI population with creatinine >7 mg/dL and blood urea nitrogen >120 mg/dL  No clear definition of RRT criteria, unclear randomization method |
| Durmaz/2003  [13] | Turkey | Post–cardiac surgery | RCT | 44/21/23 | Dialysis prophylactics | Dialysis only with acute renal failure | Prophylactic dialysis reduced post–cardiac surgery mortality | Not based on acute kidney injury population study |
| Payen/2009  [14] | France | Sepsis with organ failure | RCT | 76/37/39 | Identified focus on infection + sepsis (SIRS criteria) and one or more sepsis-induced organ failures within 24 h | No renal replacement therapy, standard sepsis management, unless renal failure and traditional criteria for RRT | Early dialysis group had worse survival outcome | Not based on acute kidney injury population study |
| Combes/2015  [15] | USA | Post–cardiac surgery, shock with high-dose catecholamine | RCT | 224/112/112 | Early high-volume HF as soon as possible after randomization | Standard care and RRT if creatinine >4 mg/dL, increase creatinine × 3 or urine output <0.3 mL/kg/h/24 h or urea >36 mmol/L or life-threatening hyperkalemia | Early high-volume HF not reduced mortality or ventilator use | Not based on acute kidney injury population study |
| Elseviers/2010  [30] | Belgium | SHARF4 study, ICU admission, AKI with serum creatinine >2 mg/dL | PC | 1303/653/650 | Discretion of the responsible physician | Conservative treatment, no RRT | Early RRT group had high mortality | Late group did not receive RRT |
| Christiansen/2017 [29] | Denmark | All patients aged 15 y or older who were treated with continuous RRT in ICU | PC | 1213/621/592 | RRT initiated at KDIGO AKI stage 2 or below, including patients not meeting the AKI criteria | KDIGO AKI stage 3 at time of RRT initiation | Early group had higher 90-d mortality  5-y mortality, ESRD, and CKD risk: no difference | Included patients not meeting AKI criteria |
| Dlamini/2017  [32] | South Africa | Fulfill KDIGO AKI, aged 13 y or older | PC | NR | Time (d) to dialysis from AKI onset: early (1 d), delayed (2–5 d) | Time (d) to dialysis from AKI onset: late (>5 d) | Sepsis and late group had higher mortality risk | No actual event number, no baseline data, and disease severity and AKI criteria in different groups |
| Gaudry/2018 [33] | France | AKIKI study protocol but only medical population (ARDS and septic shock) | RCT | 555/278/277 | RRT within 6 h after diagnosis of KDIGO stage 3 AKI | Urea >112 mg/dL, K^+^ >6 mmol/L, pH <7.15, acute pulmonary edema, oliguria/anuria for 72 h | No difference associated with mortality, MV days; more renal function recovery in late group | Duplication cohort |
| Meersch/2018  [31] | Germany | ELAIN study protocol | RCT | 230/111/119 | RRT within 8 h after diagnosis of KDIGO stage 2 AKI | RRT within 12 h after diagnosis of KDIGO stage 3 AKI or absolute indication for RRT and organ edema in the presence of AKI resistant to diuretic treatment (one attempt with loop diuretics prior to randomization) | Early group had better 1-y mortality and RRT dependence | This is the only RCT focusing on 1-y survival and is based on ELAIN study protocol (duplication) |

Abbreviation: ARDS: acute respiratory distress syndrome, AKI: acute kidney injury, HF: hemofiltration, KDIGO: Kidney Disease: Improving Global Outcomes, NR: not reported, PC: prospective cohort, RCT: randomized controlled trial, RRT: renal replacement therapy, SIRS: systemic inflammatory response syndrome

**Table S6. Newcastle–Ottawa Scale assessment of included prospective cohort studies**

| Study | Selection | | | | Comparability | Outcomes | | | Total score |
| --- | --- | --- | --- | --- | --- | --- | --- | --- | --- |
|  | Representativeness of exposed cohort | Selection of nonexposed cohort | Ascertainment of exposure | Outcome not present at the start of the study |  | Assessment of outcomes | Length of follow- up | Adequacy of follow- up |  |
| Liu,  2006 | * | * | * | * |  | * | * | * | 7 |
| Bagshaw, 2009 | * | * | * | * |  | * |  | * | 6 |
| Iyme,  2009 | * | * | * | * |  | * |  | * | 6 |
| Sabater,  2009 | * | * | * | * |  | * | * | * | 7 |
| Shiao,  2009 | * | * | * | * |  | * | * | * | 7 |
| Jun,  2014 | * | * | * | * |  | * | * | * | 7 |
| Vaara ,  2014 | * | * | * | * |  | * | * | * | 7 |
| Crescenzi,  2015 | * | * | * | * |  | * |  | * | 6 |
| Lim,  2015 | * | * | * | * |  | * | * | * | 7 |
| Park,  2016 | * | * | * | * | * | * |  | * | 7 |

A study can be awarded a maximum of one star for each numbered item within the Selection and Outcome categories. A maximum of two stars can be given for Comparability.

**Table S7. Summary of previous meta-analyses evaluating the effect of timing of RRT initiation on patient outcomes**

| Meta-analysis | Enrolled study | Primary outcome | Secondary outcome |
| --- | --- | --- | --- |
| Fayad 2018 [5] | 5 RCTs (1084 participants) | No difference in mortality (early group had trend of reducing 30-d death and death after 30 d ) | 1. Early group increased number of adverse events (RR, 1.10; 95% CI, 1.03 to 1.16)  2. Early group had trend of reducing ICU LOS, improving renal recovery |
| Cui 2018 [6] | Post–cardiac surgery AKI  4 RCTs (355 participants) | No difference in mortality | 1. No difference in ICU or hospital LOS |
| Feng 2017 [7] | 9 RCTs (1636 participants) | No difference in mortality | 1. No difference in ICU or hospital LOS |
| Lai 2017 [8] | 9 RCTs (1627 participants) | No difference in mortality | 1. No difference in ICU or hospital LOS, RRT dependence  2. Subgroup analysis: surgical or receiving CRRT population had better in-hospital survival |
| Yang 2017 [9] | 9 RCTs (1636 participants) | No difference in mortality | 1. No difference in ICU or hospital LOS, RRT dependence, RRT times, MV times, RRT-related complication |
| Wierstria 2016 [10] | 7 RCTs, 10 prospective studies, 19 retrospective studies (9 high quality with 1042 participants) | No difference in mortality | 1. No difference in ICU LOS  2. Subgroup analysis: no difference in patient population (surgical or medical) |
| Liu 2014 [11] | Post–cardiac surgery  2 RCTs, 9 retrospective studies (841 participants) | Early initiation reduced 28-d mortality | Early initiation reduced ICU LOS |

Abbreviations: AKI: acute kidney injury, CI: confidence interval, LOS: length of stay, PC: prospective cohort, RCT: randomized controlled trial, RR: relative risk, RRT: renal replacement therapy

**Table S8A. Sensitivity analysis of binary outcomes of watchful waiting strategy**

| Outcome | No. of  studies | No. of event/No. of patient, Early group | No. of event/No. of patient, Late group | Pooled odds ratio  (95% CI) | I^2^ (%) |
| --- | --- | --- | --- | --- | --- |
| Death | 6 | 407/877 | 413/887 | 1.00 (0.75 to 1.32) | 43 |
| RRT dependence | 6 | 43/877 | 56/887 | 0.79 (0.52 to 1.19) | 0 |
| Renal recovery | 3 | 157/272 | 153/285 | 1.13 (0.57 to 2.22) | 69.7 |

Abbreviation: RRT: renal replacement therapy

**Table S8B. Sensitivity analysis of continuous outcomes of watchful waiting strategy**

| Outcome | No. of studies | No. of patient, Early group | No. of patient, Late group | Pooled mean Difference (95% CI) | I^2^ (%) |
| --- | --- | --- | --- | --- | --- |
| ICU LOS | 5 | 775 | 781 | 0.17 (-0.95 to 1.29) | 0 |
| Hospital LOS | 5 | 775 | 781 | -1.57 (-3.61 to 0.46) | 0 |
| MV days | 4 | 727 | 729 | -1.09 (-2.78 to 0.60) | 29.7 |
| RRT days | 5 | 829 | 835 | 0.45 (-1.47 to 2.37) | 74.5 |

Abbreviation: LOS: length of stay, MV: mechanical ventilation, RRT: renal replacement therapy

**Table S9. Summary of findings and quality assessment through the GRADE system**

|  | | | | | | |
| --- | --- | --- | --- | --- | --- | --- |
| Early initiation of RRT compared with late initiation of RRT for Acute kidney injury | | | | | | |
| Patient or population: Acute kidney injury  Setting: Adult acute kidney injury  Intervention: Early initiation of RRT  Comparison: Late initiation of RRT | | | | | | |
| Outcomes | Anticipated absolute effects* (95% CI) | | Relative effect (95% CI) | No. of participants  (studies) | Certainty of evidence (GRADE) | Comments |
|  | Risk with late initiation of RRT | Risk with early initiation of RRT |  |  |  |  |
| Mortality follow-up: range 14–90 d | 463 per 1000 | 432 per 1000 (347 to 522) | OR 0.884 (0.616 to 1.267) | 1938 (9 RCTs) | ⨁⨁◯◯ Low^a,b^ | Weak |
| RRT dependence follow-up: range 28–90 d | 68 per 1000 | 51 per 1000 (35 to 75) | OR 0.74 (0.49 to 1.11) | 1804 (7 RCTs) | ⨁⨁◯◯ Low^a,c^ | Weak |
| Renal recovery | 513 per 1000 | 596 per 1000 (441 to 732) | OR 1.40 (0.75 to 2.59) | 731 (6 RCTs) | ⨁◯◯◯ Very low^a,b,c^ | Weak |
| ICU LOS |  | Mean ICU LOS in the intervention group was 0.09 d higher (1.01 lower to 1.19 higher) | — | 1662 (6 RCTs) | ⨁⨁◯◯ Low^a,e^ | Weak |
| MV days |  | Mean MV days in the intervention group was 3.98 d lower (7.81 lower to 0.15 lower) | — | 1602 (6 RCTs) | ⨁◯◯◯ Very low^a,d^ | Weak |
| Hospital LOS |  | Mean hospital LOS in the intervention group was 1.76 d lower (3.77 lower to 0.25 higher) | — | 1662 (6 RCTs) | ⨁⨁◯◯ Low^a,e^ | Weak |
| RRT times |  | Mean RRT time in the intervention group was 2.85 d lower (6.07 lower to 0.36 higher) | — | 1704 (6 RCTs) | ⨁◯◯◯ Very low^a,d^ | Weak |
| *The risk in the intervention group (and its 95% confidence interval) is based on the assumed risk in the comparison group and the relative effect of the intervention (and its 95% CI).  Abbreviations: CI: confidence interval; OR: odds ratio; MD: mean difference, LOS: length of stay, MV: mechanical ventilation, RRT: renal replacement therapy Explanations a. Possible performance bias in all studies due to study design in nature and selection bias in two trial (Bouman 2002; Sugahara 2004)  b. Moderate heterogeneity (different patient study group, different early or late criteria and different RRT modality)  c. Different RRT dependence or RRT free/renal recovery criteria  d. Soft outcome, not direct related to patient prognosis | | | | | | |

Abbreviations: LOS: length of stay, MV: mechanical ventilation, RRT: renal replacement therapy

**Table S10A. Trial Sequential Analysis of binary outcomes of watchful waiting strategy**

| Estimated effect | Control event proportion | Amount of heterogeneity | RR (Adjusted 95% CI) | Required information size | Monitoring boundary crossed ? | Futility boundary crossed ? | Interpretation |
| --- | --- | --- | --- | --- | --- | --- | --- |
| Mortality, accrued sample size n = 1764 | | | | | | | |
| 25% RRR | 46.5% mortality | D^2^=55% | 0.99 (0.86-1.14) | 1240, reached | Not crossed | Crossed | 25% RRR unlikely conclusively |
| 20% RRR |  |  |  | 1950, not reached | Not crossed | Crossed | 20% RRR unlikely |
| 15% RRR |  |  |  | 3486, not reached | Not crossed | Crossed | 15% RRR unlikely |
| 10% RRR |  |  |  | 7901, not reached | Not crossed | Not crossed | Inconclusive |
| 5% RRR |  |  |  | 31622, not reached | Not crossed | Not crossed | Inconclusive |
| 1% RRR |  |  |  | 779602, not reached | Not crossed | Not crossed | Inconclusive |
| RRT dependence, sample size n = 1764 | | | | | | | |
| 40% RRR | 6.3 % RRT dependence | D^2^=0 % | 0.80 (0.55-1.18) | 2367, not reached | Not crossed | Crossed | 40% RRR unlikely |
| 30% RRR |  |  |  | 4455, not reached | Not crossed | Not crossed | Inconclusive |
| 25% RRR |  |  |  | 6548, not reached | Not crossed | Not crossed | Inconclusive |
| 20% RRR |  |  |  | 10577, not reached | Not crossed | Not crossed | Inconclusive |
| Renal recovery, sample size n= 557 | | | | | | | |
| -30% RRR | 26.2 % renal recovery | D^2^=88% | 1.07 (0.93-1.23) | 8771, not reached | Not crossed | Not crossed | Inconclusive |
| -20% RRR |  |  |  | 19232, not reached | Not crossed | Not crossed | Inconclusive |
| -10% RRR |  |  |  | 74774, not reached | Not crossed | Not crossed | Inconclusive |
| -7% RRR |  |  |  | 136718, not reached | Not crossed | Not crossed | Inconclusive |

Abbreviation: RRT: renal replacement therapy, RR: relative risk, RRR: relative risk reduction.

**Table S10B. Trial Sequential Analysis of continuous outcomes of watchful waiting strategy**

| Estimated effect | Amount of heterogeneity | Pooled mean difference (Adjusted 95% CI) | Required information size | Monitoring boundary crossed ? | Futility boundary crossed ? | Interpretation |
| --- | --- | --- | --- | --- | --- | --- |
| ICU days, accrued sample size n = 1556 | | | | | | |
| 1.5 | D^2^=0% | 0.17 (-0.95 to 1.29) | 1775, not reached | Not crossed | Crossed | Mean difference 1.5 ICU days unlikely |
| 1 |  |  | 3993, not reached | Not crossed | Not crossed | Inconclusive |
| 0.5 |  |  | 15970, not reached | Not crossed | Not crossed | Inconclusive |
| 0.17 |  |  | 138147, not reached | Not crossed | Not crossed | Inconclusive |
| Hospital LOS, accrued sample size n = 1556 | | | | | | |
| -3 | D^2^=0% | -1.58 (-3.62 to 0.45) | 2923, not reached | Not crossed | Not crossed | Inconclusive |
| -2.5 |  |  | 4209, not reached | Not crossed | Not crossed | Inconclusive |
| -2 |  |  | 6577, not reached | Not crossed | Not crossed | Inconclusive |
| -1.58 |  |  | 10509 not reached | Not crossed | Not crossed | Inconclusive |
| MV days, accrued sample size n = 1456 | | | | | | |
| -2.5 | D^2^=37% | -1.1 (-2.89 to 0.59) | 2923, not reached | Not crossed | Crossed | Mean difference 2.5 MV days unlikely |
| -2 |  |  | 2144, not reached | Not crossed | Not crossed | Inconclusive |
| -1.5 |  |  | 3813, not reached | Not crossed | Not crossed | Inconclusive |
| -1.1 |  |  | 7088 not reached | Not crossed | Not crossed | Inconclusive |
| RRT days, accrued sample size n = 1664 | | | | | | |
| 3 | D^2^=89% | 0.42 (-1.53 to 2.36) | 2868, not reached | Not crossed | Crossed | Mean difference 3 RRT days unlikely |
| 2 |  |  | 6446, not reached | Not crossed | Not crossed | Inconclusive |
| 1 |  |  | 25759, not reached | Not crossed | Not crossed | Inconclusive |
| 0.42 |  |  | 148475 not reached | Not crossed | Not crossed | Inconclusive |

Abbreviation: LOS: length of stay, MV: mechanical ventilation, RRT: renal replacement therapy.

**Table S11. Summary of different RRT modalities, dialyzers, and RRT dosages in randomized controlled studies over time**

| Author/year | RRT modality | Dialyzer | Anticoagulation | Dosage or RRT prescription |
| --- | --- | --- | --- | --- |
| Bouman/2002 [28] | HF | Cellulose triacetate hollow fiber | Heparin | Early high volume: 72 L/d  Early low volume: 24–36 L/d, late: 24–36 L/d |
| Sugahara/2004 [12] | CVVHD | Polysulfone fiber | Nafamostat mesilate | Dialysate flow rate of 1 L/h, then adjusted according to clinical condition |
| Jamale/2013 [54] | iHD | Polysulfone low-flux hollow-fiber dialyzer | Heparin | Blood flow of 200–300 mL/min and dialysate flow of 500 mL/min with intermittent hemodialysis frequency over alternate days, with each session lasting 4 h |
| Wald/2015 [48] | iHD/CRRT/SLED | Dialyzer routine used in each study site | Heparin or regional citrate or none | Cardiovascular component of the Sequential Organ Failure Assessment score (SOFA_CV_) was ≥2; SLED or CRRT <2; SOFA_CV_ <2 iHD  CRRT dosage: ≥25 mL/kg/h  SLED: 3 times/wk, blood flow: 200–300 mL/min, dialysate flow: 200–400 mL/min, 8 h each session  iHD 3 times/wk, blood flow: 200–400 mL/min, dialysate flow: 500–800 mL/min, 3 h each session |
| Gaudry/2016 [55] | iHD/CRRT | NR, based on the national guideline [72] | NR, based on the national guideline [72] | NR, based on the national guideline [72]:  iHD: (1) 3 sessions per wk of ≥4 h with blood flow >200 mL/min and dialysate flow >500 mL/min, or (2) a *Kt*/*V* index >3.9/wk, or (3) maintenance of a predialysis urea concentration of 20–25 mmol/L  CRRT: minimum delivered dose 20–25 mL/kg/h |
| Zarbock/2016 [56] | CVVH | NR | Regional citrate | Prescribed effluent dosage at the time of randomization: 30 mL/kg/h |
| Barbar/2018 [57] | iHD/CVVHD/CVVH/SLED | NR, based on the national guideline [26,73] | Heparin or low-molecular-weight heparin or regional citrate | Intermittent techniques: blood flow rate 150–250 mL/min, dialysate flow rate of 300–500 mL/min, length of sessions ≥4 h and preferably ≥6 h, frequency of sessions had to be at least once every 48 h or more. Target optimal metabolic control: urea concentration <30 mmol/L or a stable salt and water balance  CRRT: minimum delivered dose 20–25 mL/kg/h |
| Lumlertgul/2018 [58] | CVVH/PIRRT/iHD | CVVH: high-flux hemofilters (AN69 or HF12) | Regional citrate (first choice), then heparin or none | Initially all RRT started with CVVH  CVVH: replacement fluid minimal 25– 30 mL/kg/h |
| Srisawat/2018 [59] | CVVH | High-flux hemofilters (AN69) | NR, based on the national guideline [26] | CVVH: replacement fluid minimal 25 mL/kg/h |

Abbreviations: CRRT: continuous renal replacement therapy, CVVH: continuous venovenous hemofiltration, CVVHD: continuous venovenous hemodialysis, iHD: intermittent hemodialysis, NR: not reported, RRT: renal replacement therapy, PIRRT: prolonged intermittent renal replacement therapy, SLED: sustained low-efficiency daily dialysis, SLED-f: sustained low-efficiency diafiltration, TIN: tubulointerstitial nephritis, UOP: urine output
