# Supplementary material for: Comparison between watchful waiting strategy and early initiation of renal replacement therapy in the critically ill acute kidney injury population: an updated systematic review and meta-analysis
Source: Ann Intensive Care. 2020 Mar 3;10:30. doi: 10.1186/s13613-020-0641-5 (PMC7054512; doi:10.1186/s13613-020-0641-5)
Supplement: Supplementary file 2 — Additional file 2. Additional documents. [file 13613_2020_641_MOESM2_ESM.docx]

**Supplemental Document 1. Definition of early versus late RRT initiation.**

Most of the included RCTs used the period after AKI diagnosis or fulfilled enrollment criteria as the early criteria (ranging from 6 to 12 h). Two of the 9 RCTs used other definitions for early RRT (Sugahara, 2004, used UOP < 30 mL/h for 3 h or daily urine output < 750 mL/d and Jamale, 2013, used urea >70 mg/dL or creatinine >7 mg/dL). Eight of the 9 RCTs used conventional indication for RRT as the late criteria or as a part of the late criteria. One of the RCTs (Sugahara, 2004) used urinary output (<20 mL/h for 2 h or daily urine output < 500 mL/d) as the cutoff point for the late group. Four of the aforementioned 9 RCTs used conventional indication only (Bouman, 2002; Jamale, 2013; Wald, 2015; Lumlertgul, 2018). Others used mixed criteria for late RRT initiation. Despite conventional indication, the AKIKI study started RRT in the late group for oliguria/anuria at 72 hours. In the ELAIN trial, RRT was arranged within 12 hours in the late group with AKI progression from KDIGO stage 2 to 3 without need to fulfill conventional indication. The IDEAL-ICU trial also started RRT in the late group without renal recovery (defined by urine output) after 48 hours of AKI diagnosis, which was shorter than that in the AKIKI trial (72 hours) but longer than that in the ELAIN trial (within 12 h after KDIGO stage 3). EARLY-RRT trials started RRT in the late group without oliguria and anuria, but no definite duration was mentioned. The early and late criteria in prospective studies were more diverse, and nearly half of them used the median level of urea or creatinine as cutoff points. Two studies used the RIFLE stage as the cutoff point (Sabater, 2009; Shiao, 2009). One used conventional indication as late criteria (Vaara, 2014).

**Supplemental Document 2. Bias assessment and outcomes of 10 prospective cohort studies**

**Assessment of risk of bias**

We assessed the risk of bias of the included prospective studies by using the Newcastle-Ottawa Scale (NOS) online [68]. The NOS comprises 3 subscales (a total of 8 items within 3 subscales), and the maximum score is 9. A study with a total score of less than or equal to 7 is considered as a low-quality study as previous similar study [10]. The result of bias assessment of prospective cohort studies is summarized in **Supplemental Table 6.**

**Study characteristics**

Total 10 prospective cohort studies included 3269 patients with AKI were enrolled. Most of then enrolled mixed surgically or medically critical illness patients.

**Primary outcomes**

The pooled odds ratio of death in prospective cohort studies was 0.67 (95% CI: 0.44 to 1.02)


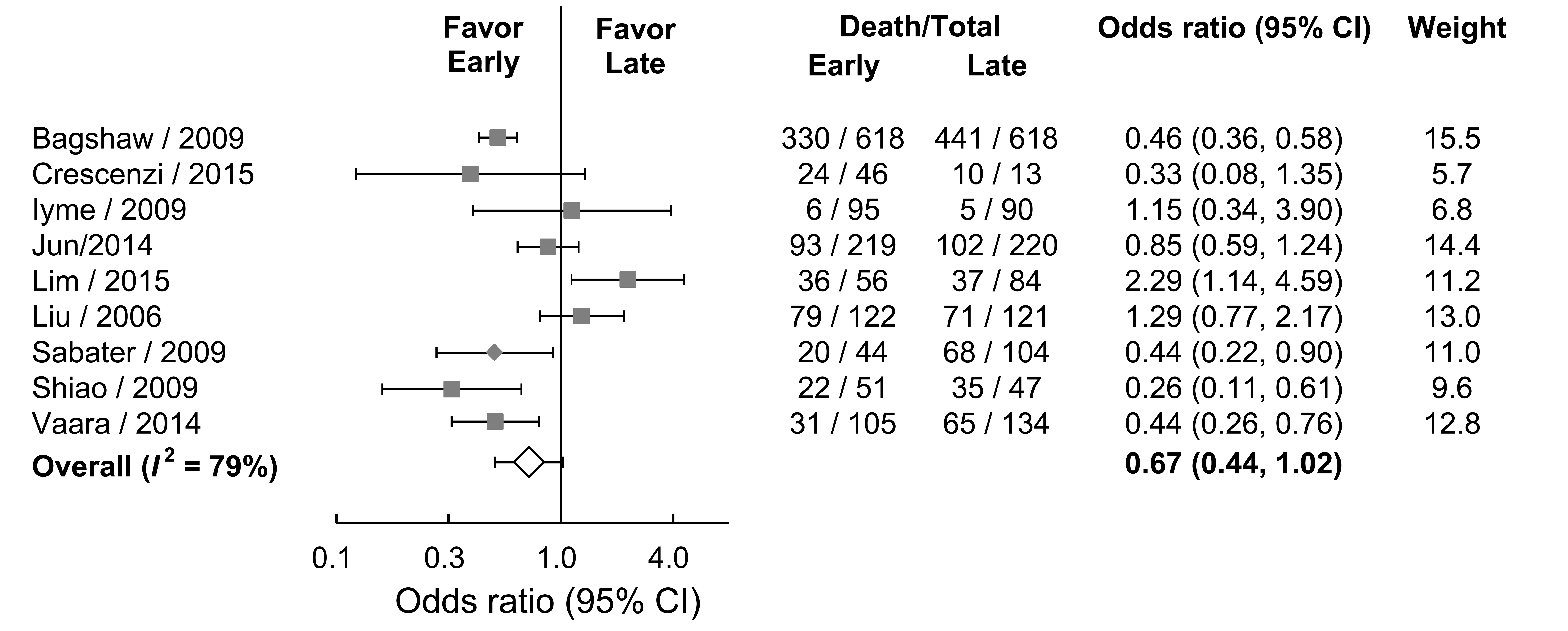


**Secondary outcomes**

The mean difference in ICU LOS was nearly neutral: -0.31 days (95% CI: -3.05 to 2.44 days) in prospective studies and the hospital LOS was not significantly different between the groups in either group (-0.43 days, 95% CI: -11.50 to 10.73 days). Only one enrolled prospective study had reported outcome of MV (Crescenzi, 2015). Regarding to RRT days, no difference was noted between the early and late groups in prospective studies (0.07 days, 95% CI: -1.48 to 1.63 days). The pooled odds ratio showed no significant difference between the groups in terms of renal recovery (OR: 2.14, 95% CI: 0.52 to 8.86)


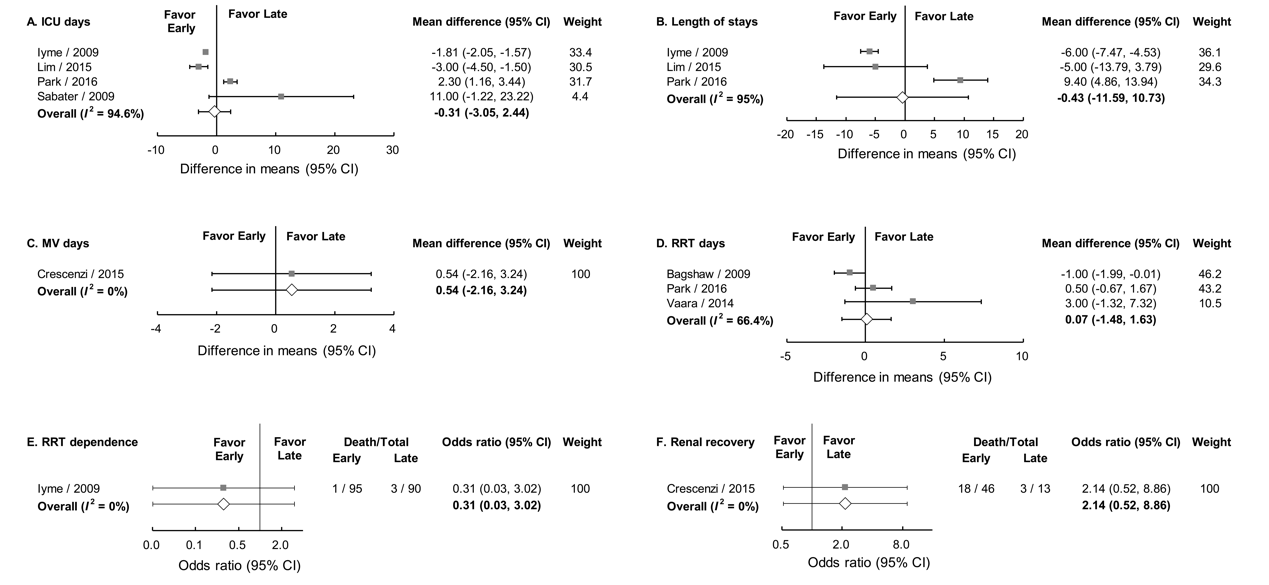


**Supplemental Document 3. Detailed explanation of modality of RRT and definition of acute kidney injury for the included studies.**

The modality of RRT varied in studies, and 10 of them utilized a mixture of continuous renal replacement therapy (CRRT), intermittent hemodialysis (iHD), or sustained low efficiency dialysis (SLED). CRRT was the only modality of RRT in 7 trials. Hemofiltration was the choice of modality in one old study (Bouman, 2002). A summary of other fundamental characteristics of all evaluated trails is provided in Table 1, Table 2, and Supplemental Table 2. Six of 10 RCTs (Jamale, 2013; Wald, 2015; Gaudry, 2016; Zarbock, 2016; Barbar, 2018; Lumlertgul, 2018; Srisawat 2018) that fulfilled our predefined criteria were identified to conduct analysis to delineate whether the wait and see strategy and using conventional RRT indication are still safe.

The definition of acute kidney injury also varied in the included trials. Four of the 9 RCTs used predefined criteria (Bouman, 2002; Sugahara, 2004; Jamale, 2013; Wald, 2015), three of the 9 RCTs used KDIGO criteria (Gaudry, 2016; Zarbock, 2016; Lumlertgul, 2018), and two used RIFLE criteria (Barbar, 2018; Srisawat, 2018). Most RCTs except one (Sugahara 2004, late criteria; urine output <20 mL/h for 2 h or daily urine output <500 mL/d) used conventional indication for RRT initiation as the late criteria or a part of late criteria (hyperkalemia, range: 6.0-6.5 mmol/L, fluid overload with/without organ edema, metabolic acidosis with pH < 7.15-7.20, uremia with different blood urea cutoff point) (**Supplemental Table 2**). The late criteria in prospective trials varied more, and most of them used median levels of urea, creatinine, and urine output as cutoff point for early or late initiation of RRT (**Supplemental** **Table 3**). Four studies used high plasma NGAL as enrollment criteria (Wald, 2015; Zarbock, 2016; Lumlertgul, 2018; Srisawat, 2018), and the cutoff point of plasma NGAL level ranged from 150 to 400 ng/mL.

**Supplemental Document 4. Detailed explanation of risk of bias assessments in the included randomized controlled trials.**

**Allocation and random sequence generation (Selection bias)**

The method of allocation and random sequence generation was not provided in two studies (Bouman, 2002; Sugahara, 2004). Other studies were assessed as low risk regarding allocation and random sequence generation.

**Blinding (Performance and detection bias)**

All studies were assessed as low risk in the domain of detection bias because the outcome measured was unlikely to be affected. The blinding of participants and personnel were all assessed as unclear because of the study’s interventional design and insufficient information for judgement.

Incomplete outcome data (Attrition bias)

This domain was assessed as low risk in most trials except one (Sugahara, 2004). Twelve of forty patients randomized were not included in final analysis and we assessed it as high attrition bias.

Selective reporting (Reporting bias)

Two trials (Bouman, 2002; Sugahara, 2004) were assessed as unclear risk in this domain. Both studies did not provide information about prespecified way for outcome measurement or outcome definition or protocol. No definition of renal recovery in Bouman 2002 was mentioned in the article.

Other bias

All studies were considered as low risk in this domain.

**Supplemental Document 5. Detailed discussion of previous meta-analyses**

Older review articles were based mostly on retrospective or prospective studies [36-37], and possible survival advantage from early RRT initiation was observed. Several possible limitations should be considered like not based on AKI population. For example, prophylactic RRT have survival advantage demonstrated by Durmaz et al. in post cardiac surgery patients [13] but this result is not supported by HEROICS study [15]. Prophylactic RRT in patients with sepsis and organ failure may even result in poor outcomes [14]. Report from a 1-year follow-up of ELAIN trials demonstrated a positive effect of early RRT initiation on mortality and renal recovery in surgical patients but not supported by recent meta-analyses which might result from the difference in RRT modality, as observed by Schneider et al [38] and different underlying condition [39]. Despite these numerous studies and reviews thus far, the answer to this long debating question is still elusive.

**Supplemental Document 6. Detailed discussion of contradictory results from prospective cohort studies and RCTs**

Similarly, several confounder should be discussed in the study by Sugahara et al., including attribution bias (30% patients received randomization; not included in outcome analysis), relatively short follow-up duration (14 days), and extremely high mortality in the late arm (14% vs. 86%). Several articles discussed the different results from AKIKI and ELAIN trials. As Shiao et al. [42] and other authors [43-44] pointed out, in comparison to the AKIKI trial, the ELAIN trial had possible selection and performance bias (single center), more nonrenal organ failure, an earlier AKI stage than the AKIKI trial, with NGAL as the enrollment criterion, less sepsis, only surgical patients (compared with the AKIKI trial with 80% medical septic patients), CRRT as the initial RRT modality, and more severe fluid overload with pulmonary edema, which require further discussion. The enrollment criteria for the ELAIN trial included refractory fluid overload (worsening pulmonary edema, PaO2/FiO2 < 300 mmHg, or fluid balance >10% of body weight). Substantial research has indicated that fluid overload is a poor prognostic factor, especially fluid accumulation of more than 10% of body weight [45-47], which may contribute to the survival and other secondary outcome benefits observed from the early RRT arm in the ELAIN trial.

Several included prospective studies showed positive (Bagshaw, 2009; Sabater, 2009; Shiao, 2009; Vaara, 2014) or negative result (Lim, 2015) of early RRT. Some possible explanations for these contradictory results in prospective cohort studies are as follows: 1) different disease severities in among two groups, 2) the cutoff point for the early and late arm was determined based on the median biochemical value, 3) most trials did not exclude the emergent need for RRT before enrolling patients into trials.

**Supplemental Document 7. Possible benefit of early RRT in surgical population and result of trial sequence analysis**

There are possible benefit from early RRT initiation included shorter MV days in overall enrolled population and RRT time in surgical population. One explanation is that the early RRT group had more favorable fluid balance control, which may help to shorten the MV-dependence days and aid in more effective renal recovery. Fluid overload results in kidney edema which could lead to lower renal perfusion, poor prognosis [49-50]. We detected possible benefit from early RRT initiation in surgical population which should be interpreted carefully . In contrast to surgical population, these benefit were less likely to detected in medical population. Septic AKI population were prone to have unstable hemodynamic and higher mortality [51]. Post hoc analysis from AKIKI trials focused on ARDS or septic shock patients found no benefit from early RRT [33]. Notably, with adequate bicarbonate therapy in selective AKI patients with metabolic acidosis, RRT might be avoided by recent trial [52].

However, only one study (ELAIN, 2016) that focused on the pure surgical population reported data regarding MV and RRT time.

Trial sequence analysis was used for examining one significant secondary outcome (shorter MV days in early RRT group) by conventional meta-analysis of enrolled RCTs. We used the α = 0.05 (two- sided) and β = 0.20 (power 80%) which leading to statistical significant level of 5% and 95% CIs for MV days. An estimated mean difference of MV days of 3.9 days was applied. The accrued information size (n= 1602) has not yet reach required information size line (n = 3044) to confirm a mean 3.9 days reduction of MV days. The Z line only crossed the traditional boundary but not crossed monitoring efficacy line nor the futility line to exclude possible benefit **(Additional file 5: Figure S3)**. Therefore, type 1 error should be considered due to possible premature conclusion from our meta-analysis. More trials are needed for further confirmation.

**Supplemental Document 8. Trial sequence analysis of outcomes of watchful waiting strategy**

We concluded that watchful waiting strategy is safe by conventional meta-analysis. We then performed trial sequence analysis with an alpha level of 5%, power of 80%, and an estimated event proportion (mortality) at control arm of 46.5%. The Z curve crossed the futility line with an estimated RRR 15% or higher but not reached requiring information sample size line (n=3486) with accrued sample (n=1764). The required information sample size was reached if using an estimated RRR 25%. Therefore, we may said that with current evidence, watchful waiting strategy is safe and early RRT unlikely brings a mortality risk reduction of 15% but smaller benefit effect could not be excluded owing to relative small accrued sample size (**Supplemental Table 10A ,10B** and **Additional file 6: Figure S4)** .

Besides, traditional meta-analysis showed safety of watchful waiting strategy regarding to the six secondary outcomes; however according to trial sequence analysis, the information size is relative small to make firm conclusion. (**Supplemental Table 10A ,10B).** With an estimated 40% relative risk reduction of RRT dependence, current evidence showed this benefit was less likely to happen but smaller benefit could not be excluded. In regarding to renal recovery, accrued sample size was insufficiency to make firm conclusion. In considering to secondary outcome, an estimated mean difference 0.17 days of ICU days, estimated mean difference 1.58 days of hospital length of stays, estimated mean difference 1.1 days of MV days and estimated mean difference 0.42 days of RRT days were applied and no firm conclusion could be obtained due to relative small estimated effect from early RRT strategy and the Z line all not crossed requiring sample size line. However, the evidence was sufficient to exclude more large difference between early or late RRT groups; for example, a mean difference of 2.5 days MV days from early RRT was unlikely to happened since the futility line was crossed.
