# Supplementary figures and images for: Comparison between watchful waiting strategy and early initiation of renal replacement therapy in the critically ill acute kidney injury population: an updated systematic review and meta-analysis
Source: Ann Intensive Care. 2020 Mar 3;10:30. doi: 10.1186/s13613-020-0641-5 (PMC7054512; doi:10.1186/s13613-020-0641-5)

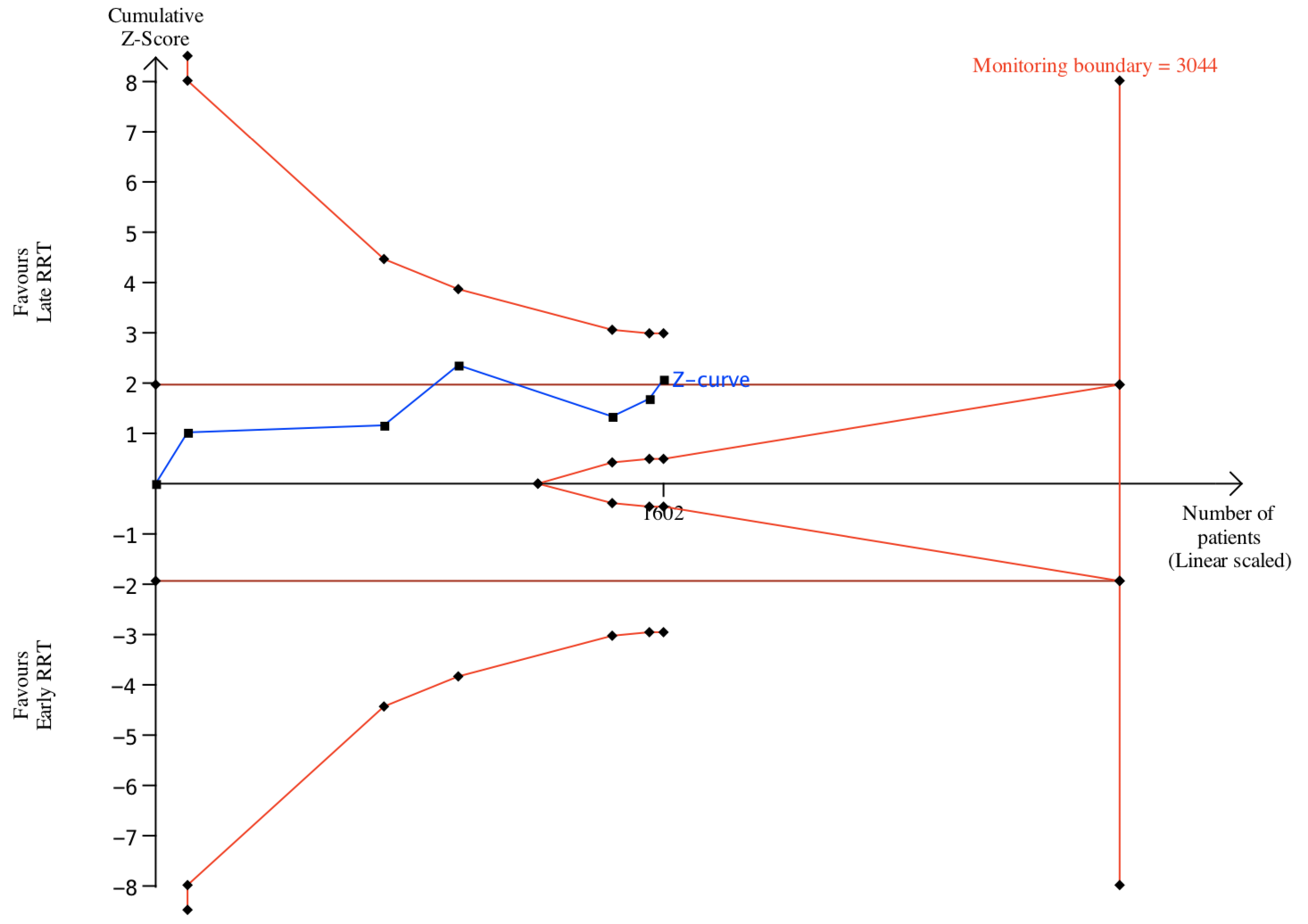

Supplement: Supplementary file 5 — Additional file 5: Figure S3. Trial Sequential Analysis of mechanical ventilation days in the included randomized controlled trials. [file 13613_2020_641_MOESM5_ESM.tif]

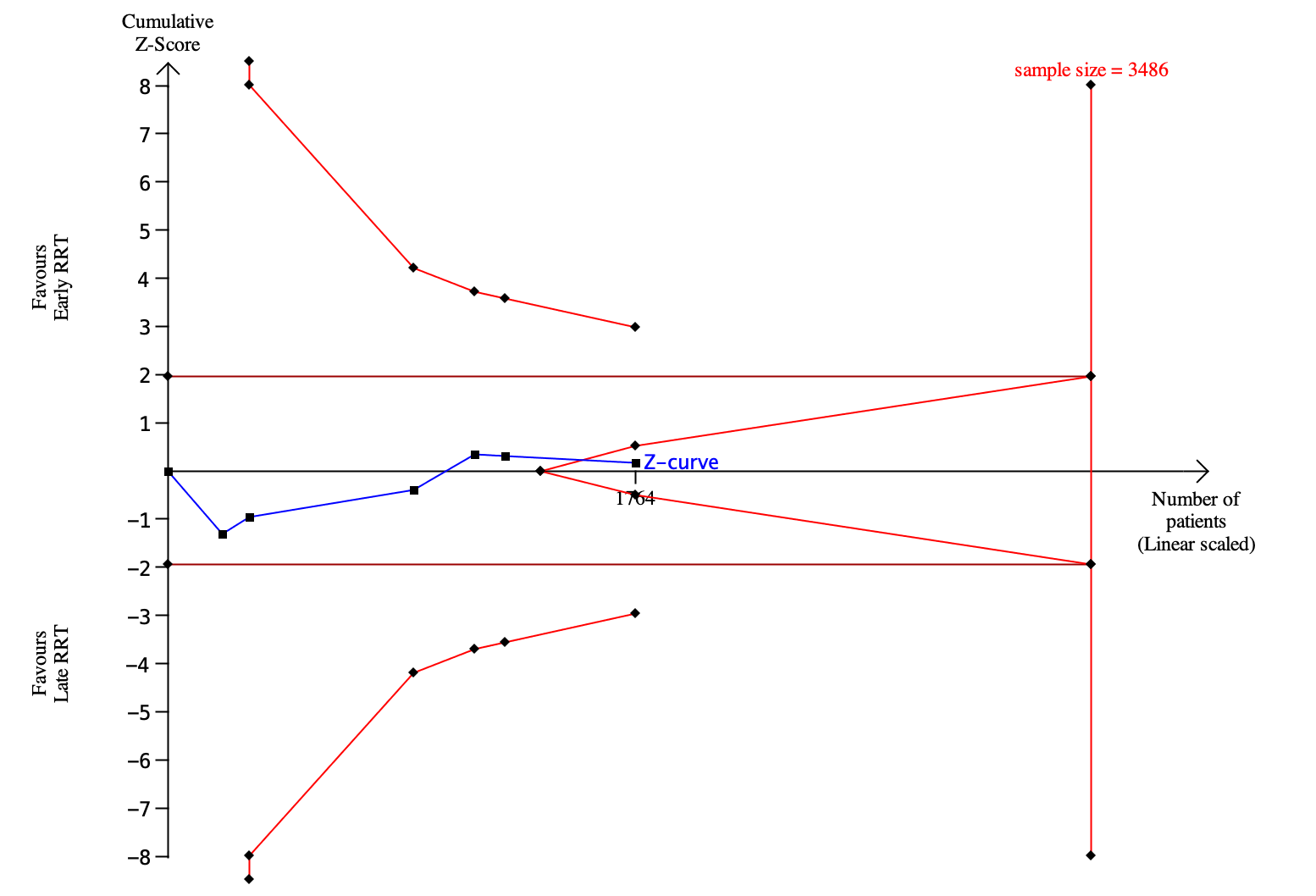

Supplement: Supplementary file 6 — Additional file 6: Figure S4. Trial Sequential Analysis of mortality with an estimated relative risk reduction of 15% between the early and late renal replacement therapy initiation groups in the six randomized controlled trials with the watchful waiting strategy. [file 13613_2020_641_MOESM6_ESM.tif]

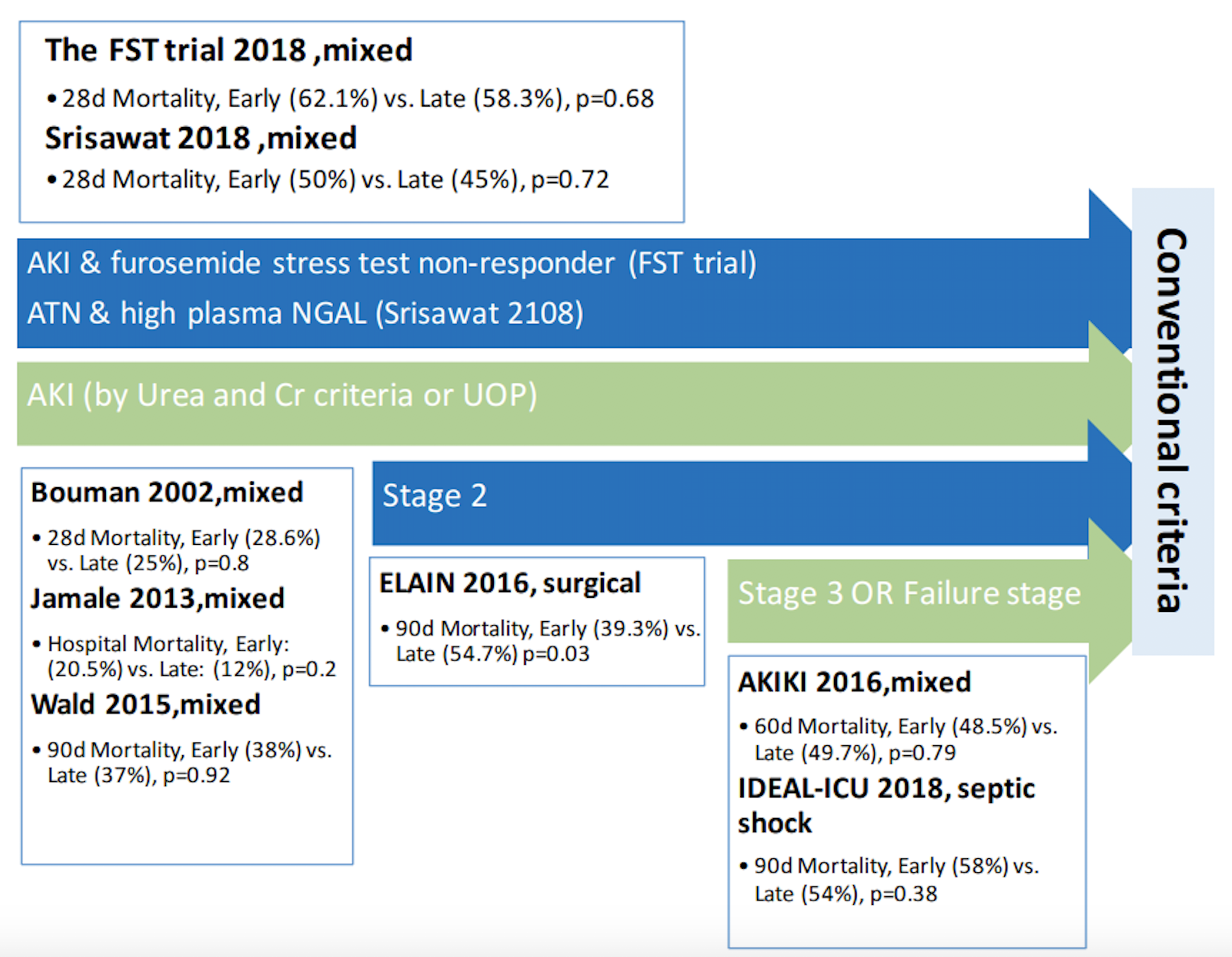

Supplement: Supplementary file 7 — Additional file 7: Figure S5. Summary of early and late renal replacement therapy initiation criteria and primary outcome of the eight randomized controlled trials using conventional criteria as late criteria. [file 13613_2020_641_MOESM7_ESM.tif]
